# Channel Identification Machines

**DOI:** 10.1155/2012/209590

**Published:** 2012-11-14

**Authors:** Aurel A. Lazar, Yevgeniy B. Slutskiy

**Affiliations:** Department of Electrical Engineering, Columbia University, New York, NY 10027, USA

## Abstract

We present a formal methodology for identifying a channel in a system consisting of a communication channel in cascade with an asynchronous sampler. The channel is modeled as a multidimensional filter, while models of asynchronous samplers are taken from neuroscience and communications and include integrate-and-fire neurons, asynchronous sigma/delta modulators and general oscillators in cascade with zero-crossing detectors. We devise channel identification algorithms that recover a projection of the filter(s) onto a space of input signals loss-free for both scalar and vector-valued test signals. The test signals are modeled as elements of a reproducing kernel Hilbert space (RKHS) with a Dirichlet kernel. Under appropriate limiting conditions on the bandwidth and the order of the test signal space, the filter projection converges to the impulse response of the filter. We show that our results hold for a wide class of RKHSs, including the space of finite-energy bandlimited signals. We also extend our channel identification results to noisy circuits.

## 1. Introduction

Signal distortions introduced by a communication channel can severely affect the reliability of communication systems. If properly utilized, knowledge of the channel response can lead to a dramatic improvement in the performance of a communication link. In practice, however, information about the channel is rarely available a priori and the channel needs to be identified at the receiver. A number of channel identification methods [1] have been proposed for traditional clock-based systems that rely on the classical sampling theorem [2, 3]. However, there is a growing need to develop channel identification methods for asynchronous nonlinear systems, of which time encoding machines (TEMs) [4] are a prime example.

TEMs naturally arise as models of early sensory systems in neuroscience [5, 6] as well as models of nonlinear samplers in signal processing and analog-to-discrete (A/D) converters in communication systems [4, 6]. Unlike traditional clock-based amplitude-domain devices, TEMs encode analog signals as a strictly increasing sequence of irregularly spaced times (*t*_*k*_)_*k*∈*ℤ*_. As such, they are closely related to irregular (amplitude) samplers [4, 7] and, due to their asynchronous nature, are inherently low-power devices [8]. TEMs are also readily amenable to massive parallelization [9]. Furthermore, under certain conditions, TEMs faithfully represent analog signals in the time domain; given the parameters of the TEM and the time sequence at its output, a time decoding machine (TDM) can recover the encoded signal loss-free [4, 5].

**Figure 1:**
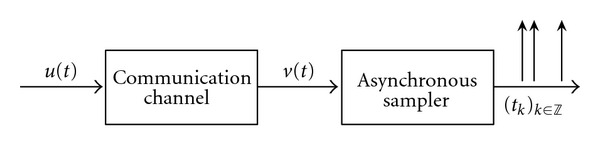
Modeling the channel identification problem. A known multidimensional signal *u*(*t*), *t* ∈ ℝ, is first passed through a communication channel. A nonlinear sampler then maps the output *v* of the channel into an observable time sequence (*t*_*k*_)_*k*∈*ℤ*_.

A general TEM of interest is shown in Figure 1. An analog multidimensional signal **u** is passed through a channel with memory that models physical communication links. We assume that the effect of this channel on the signal **u** can be described by a linear multidimensional filter. The output of the channel *v* is then mapped, or encoded, by a nonlinear asynchronous sampler into the time sequence (*t*_*k*_)_*k*∈*ℤ*_. A few examples of samplers include asynchronous A/D converters such as the one based on an asynchronous sigma/delta modulator (ASDM) [4], nonlinear oscillators such as the van der Pol oscillator in cascade with a zero-crossing detector (ZCD) [6], and spiking neurons such as the integrate-and-fire (IAF) or the threshold-and-fire (TAF) neurons [9]. The above-mentioned asynchronous samplers incorporate the temporal dynamics of spike (pulse) generation and allow one to consider, in particular for neuroscience applications, more biologically plausible nonlinear spike generation (sampling) mechanisms.

In this paper, we investigate the following *nonlinear* identification problem: given both the input signal **u** and the time sequence (*t*_*k*_)_*k*∈*ℤ*_ at the output of a TEM, what is the channel filter? System identification problems of this kind are key to understanding the nature of neural encoding and processing [10–14], process modeling and control [15], and, more generally, methods for constructing mathematical models of dynamical systems [16].

Identification of the channel from a time sequence is to be contrasted with existing methods for rate-based models in neuroscience (see [10] for an extensive review). In such models the output of the system is taken to be its instantaneous response rate and the nonlinear generation of a time sequence is not explicitly modeled. Furthermore, in order to fit model parameters, identification methods for such models typically require the response rate to be known [17]. This is often difficult in practice since the same experiment needs to be repeated a large number of times to estimate the response rate. Moreover, the use of the same stimulus typically introduces a systematic bias during the identification procedure [10].

The channel identification methodology presented in this paper employs test signals that are neither white nor have stationary statistics (e.g., Gaussian with a fixed mean/variance). This is a radical departure from the widely employed nonlinear system identification methods [10], including the spike-triggered average [18] and the spike-triggered covariance [19] methods. We carry out the channel identification using input signals that belong to reproducing kernel Hilbert spaces (RKHSs), and, in particular, spaces of bandlimited functions, that is, functions that have a finite support in the frequency domain. The latter signals are extensively used to describe sensory stimuli in biological systems and to model signals in communications. We show that for such signals the channel identification problem becomes mathematically tractable. Furthermore, we demonstrate that the choice of the input signal space profoundly effects the type of identification results that can be achieved.

The paper is organized as follows. In Section 2, we introduce three application-driven examples of the system in Figure 1 and formally state the channel identification problem. In Section 3, we present the single-input single-output (SISO) channel identification machine (CIM) for the finite-dimensional input signal space of trigonometric polynomials. Using analytical methods and simulations, we demonstrate that it is possible to identify the projection of the filter onto the input space loss-free and show that the SISO CIM algorithm can recover the original filter with arbitrary precision, provided that both the bandwidth and the order of the input space are sufficiently high. Then, in Section 4, we extend our methodology to multidimensional systems and present multi-input single-output (MISO) CIM algorithms for the identification of vector-valued filters modeling the channel. We generalize our methods to classes of RKHSs of input signals in Section 5.1 and work out in detail channel identification algorithms for infinite-dimensional Paley-Wiener spaces. In Section 5.2 we discuss extensions of our identification results to noisy systems, where additive noise is introduced either by the channel or the asynchronous sampler. Finally, Section 6 concludes our work.

## 2. The Channel Identification Problem

 We investigate a general I/O system comprised of a filter or a bank of filters (i.e., a linear operator) in cascade with an asynchronous (nonlinear) sampler (Figure 1). The I/O circuit belongs to the class of [Filter]-[Asynchronous Sampler] circuits. In general terms, the input to such a system is a vector-valued analog signal **u** = [*u* ^1^(*t*), *u* ^2^(*t*),…, *u* ^*M*^(*t*)]^*T*^, *t* ∈ ℝ, *M* ∈ *ℕ*, and the output is a time sequence (*t*_*k*_)_*k*∈*ℤ*_ generated by its asynchronous sampling mechanism. In the neural coding literature, such a system is called a time encoding machine (TEM) [4] as it encodes an unknown signal **u** into an observable time sequence (*t*_*k*_)_*k*∈*ℤ*_.

### 2.1. Examples of Asynchronous SISO and MISO Systems

**Figure 2:**
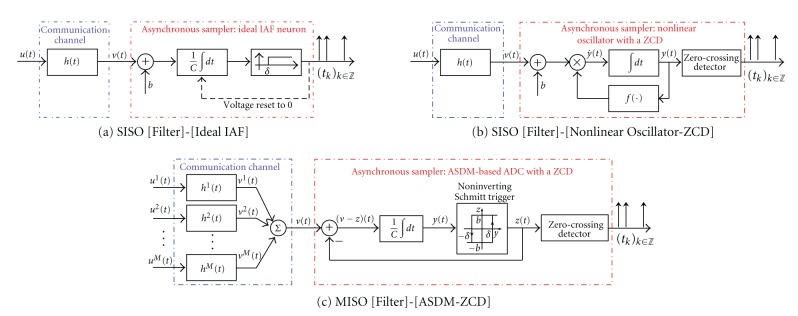
Examples of systems arising in neuroscience and communications. (a) Single-input single-output model of a sensory neuron. (b) Single-input single-output nonlinear oscillator in cascade with a zero-crossing detector. (c) Multi-input single-output analog-to-discrete converter implemented with an asynchronous sigma-delta modulator. *M* liner filters model *M* (different) communication links.

 An instance of the TEM in Figure 1 is the SISO [Filter]-[Ideal IAF] neural circuit depicted in Figure 2(a). Here the filter is used to model the aggregate processing of a stimulus performed by the dendritic tree of a sensory neuron. The output of the filter *v* is encoded into the sequence of spike times (*t*_*k*_)_*k*∈*ℤ*_ by an ideal integrate-and-fire neuron. Identification of dendritic processing in such a circuit is an important problem in systems neuroscience. It was first investigated in [20]. Another instance of the system in Figure 1 is the SISO [Filter]-[Nonlinear Oscillator-ZCD] circuit shown in Figure 2(b). In contrast to the first example, where the input was coupled additively, in this circuit the biased filter output *v* is coupled multiplicatively into a nonlinear oscillator. The zero-crossing detector then generates a time sequence (*t*_*k*_)_*k*∈*ℤ*_ by extracting zeros from the observable modulated waveform at the output of the oscillator. Called a TEM with multiplicative coupling [6], this circuit is encountered in generalized frequency modulation [21].

An example of a MISO system is the [Filter]-[ASDM-ZCD] circuit shown in Figure 2(c). Similar circuits arise practically in all modern-day A/D converters and constitute important front-end components of measurement and communication systems. Each signal *u* ^*m*^(*t*), *t* ∈ ℝ, *m* = 1,2,…, *M*, is transmitted through a communication channel and the effect of the channel on each signal is modeled using a linear filter with an impulse response *h* ^*m*^(*t*), *t* ∈ ℝ, *m* = 1,2,…, *M*. The aggregate channel output *v*(*t*) = ∑_*m*=1_^*M*^ *v* ^*m*^(*t*) = ∑_*m*=1_^*M*^(*u* ^*m*^∗*h* ^*m*^)(*t*), where *u* ^*m*^∗*h* ^*m*^ denotes the convolution of *u* ^*m*^ with *h* ^*m*^, is additively coupled into an ASDM. Specifically, *v*(*t*) is passed through an integrator and a noninverting Schmitt trigger to produce a binary output *z*(*t*)∈{−*b*, *b*}, *t* ∈ ℝ. A zero-crossing detector is then used to extract the sequence of zero-crossing times (*t*_*k*_)_*k*∈*ℤ*_ from *z*(*t*). Thus, the output of this [Filter]-[ASDM-ZCD] circuit is the time sequence (*t*_*k*_)_*k*∈*ℤ*_.

### 2.2. Modeling the Input Space

We model channel input signals *u* = *u*(*t*), *t* ∈ ℝ, as elements of the space of trigonometric polynomials *ℋ* (see Section 5.1 for more general input spaces).

**Definition 1**

The space of trigonometric polynomials *ℋ* is a Hilbert space of complex-valued functions

(1) $$\begin{array}{c} u\left(t\right)=\frac{1}{\sqrt{T}}\sum_{l=-L}^{L}u_{l}\exp\left(\frac{jl\Omega t}{L}\right),\;t\in \left[0,T\right], \end{array}$$

where *u*_*l*_ ∈ *ℂ*, *Ω* is the bandwidth, *L* is the order and *T* = 2*πL*/*Ω*, endowed with the inner product 〈·, ·〉:*ℋ* × *ℋ* → *ℂ*

(2) $$\begin{array}{c} \langle u,w\rangle =\int_{0}^{T}u\left(t\right)\bar{w(t)}dt. \end{array}$$

Given the inner product in (2), the set of elements

(3) $$\begin{array}{c} e_{l}\left(t\right)=\frac{1}{\sqrt{T}}\exp\left(\frac{jl\Omega t}{L}\right),\;\;l=-L,-L+1,\ldots ,L, \end{array}$$

forms an orthonormal basis in *ℋ*. Thus, any element *u* ∈ *ℋ* and any inner product 〈*u*, *w*〉 can be compactly written as *u* = ∑_*l*=−*L*_^*L*^ *u*_*l*_ *e*_*l*_ and $\langle u,w\rangle =\sum_{l=-L}^{L}u_{l}\bar{w_{l}}$. Moreover, *ℋ* is a reproducing kernel Hilbert space (RKHS) with a reproducing kernel (RK) given by

(4) $$\begin{array}{c} K\left(s,t\right)=\sum_{l=-L}^{L}e_{l}\left(s\right)\bar{e_{l}\left(t\right)}=\frac{1}{T}\sum_{l=-L}^{L}\exp\left(\frac{jl\Omega }{L}\left(s-t\right)\right), \end{array}$$

also known as a Dirichlet kernel [22].

We note that a function *u* ∈ *ℋ* satisfies *u*(0) = *u*(*T*). There is a natural connection between functions on an interval of length *T* that take on the same values at interval end-points and functions on ℝ that are *T*-periodic: both provide equivalent descriptions of the same mathematical object, namely a function on a circle. By abuse of notation, in what follows *u* will denote both a function defined on an interval of length *T* and a function defined on the entire real line. In the latter case, the function *u* is simultaneously periodic with period *T* and bandlimited with bandwidth *Ω*, that is, it has a finite spectral support supp⁡(*ℱu*)⊆[−*Ω*, *Ω*], where *ℱ* denotes the Fourier transform. In what follows we will assume that *u*_*l*_ ≠ 0 for all *l* = −*L*, −*L* + 1,…, *L*, that is, a signal *u* ∈ *ℋ* contains all 2*L* + 1 frequency components.

### 2.3. Modeling the Channel and Channel Identification

The channel is modeled as a bank of *M* filters with impulse responses *h* ^*m*^, *m* = 1,2,…, *M*. We assume that each filter is linear, causal, BIBO-stable and has a finite temporal support of length *S* ≤ *T*, that is, it belongs to the space *H* = {*h* ∈ *𝕃* ^1^(ℝ) | supp⁡(*h*)⊆[0, *T*]}. Since the length of the filter support is smaller than or equal to the period of an input signal, we effectively require that for a given *S* and a fixed input signal bandwidth *Ω*, the order *L* of the space *ℋ* satisfies *L* ≥ *S* · *Ω*/(2*π*). The aggregate channel output is given by *v*(*t*) = ∑_*m*=1_^*M*^(*u* ^*m*^∗*h* ^*m*^)(*t*). The asynchronous sampler maps the input signal *v* into the output time sequence (*t*_*k*_)_*k*=1_^*n*^, where *n* denotes the total number of spikes produced on an interval *t* ∈ [0, *T*].

**Definition 2**

A signal **u** ∈ *ℋ* ^*M*^ at the input to a [Filter]-[Asynchronous Sampler] circuit together with the resulting output *𝕋* = (*t*_*k*_)_*k*=1_^*n*^ of that circuit is called an input/output (I/O) pair and is denoted by (**u**, *𝕋*).

We are now in a position to define the channel identification problem.

**Definition 3**

Let (**u** ^*i*^), *i* = 1,2,…, *N*, be a set of *N* signals from a test space *ℋ* ^*M*^. A channel identification machine implements an algorithm that estimates the impulse response of the filter from the I/O pairs (**u** ^*i*^, *𝕋* ^*i*^), *i* = 1,2,…, *N*, of the [Filter]-[Asynchronous Sampler] circuit.

**Remark 4**

We note that a CIM recovers the impulse response of the filter based on the knowledge of I/O pairs (**u** ^*i*^, *𝕋* ^*i*^), *i* = 1,2,…, *N*, and the sampler circuit. In contrast, a time decoding machine recovers an encoded signal **u** based on the knowledge of the entire TEM circuit (both the channel filter and the sampler) and the output time sequence *𝕋*.

## 3. SISO Channel Identification Machines

As already mentioned, the circuits under investigation consist of a channel and an asynchronous sampler. Throughout this paper, we will assume that the structure and the parameters of the asynchronous sampler are known. We start by formally describing asynchronous channel measurements in Section 3.1. Channel identification algorithms from asynchronous measurements are given in Section 3.2. Examples characterizing the performance of the identification algorithms are discussed in Section 3.3.

### 3.1. Asynchronous Measurements of the Channel Output

 Consider the SISO [Filter]-[Ideal IAF] neural circuit in Figure 2(a). In this circuit, an input signal *u* ∈ *ℋ* is passed through a filter with an impulse response (or kernel) *h* ∈ *H* and then encoded by an ideal IAF neuron with a bias *b* ∈ ℝ_+_, a capacitance *C* ∈ ℝ_+_, and a threshold *δ* ∈ ℝ_+_. The output of the circuit is a sequence of spike times (*t*_*k*_)_*k*=1_^*n*^ on the time interval [0, *T*] that is available to an observer. This neural circuit is an instance of a TEM and its operation can be described by a set of equations

(5) $$\begin{array}{c} \int_{t_{k}}^{t_{k+1}}\left(u*h\right)\left(s\right)ds=q_{k},\;k=1,2,\ldots ,n-1, \end{array}$$

where *q*_*k*_ = *Cδ* − *b*(*t*_*k*+1_ − *t*_*k*_). Intuitively, at every spike time *t*_*k*+1_ the ideal IAF neuron is providing a measurement *q*_*k*_ of the signal *v*(*t*) = (*u*∗*h*)(*t*) on the time interval [*t*_*k*_, *t*_*k*+1_).

**Definition 5**

The mapping of an analog signal *u*(*t*), *t* ∈ ℝ, into an increasing sequence of times (*t*_*k*_)_*k*∈*ℤ*_ (as in (5)) is called the *t*-transform [4].

**Definition 6**

The operator *𝒫* : *H* → *ℋ* given by

(6) $$\begin{array}{c} \left(𝒫h\right)\left(t\right)=\int_{0}^{T}h\left(s\right)\bar{K\left(s,t\right)}ds \end{array}$$

is called the projection operator.

**Proposition 7 (conditional duality)**

For all *u* ∈ *ℋ*, a [Filter]-[Ideal IAF] TEM with a filter kernel *h* is I/O-equivalent to a [Filter]-[Ideal IAF] TEM with the filter kernel *𝒫h*. Furthermore, the CIM algorithm for identifying the filter kernel *𝒫h* is equivalent to the TDM algorithm for recovering the input signal *𝒫h* encoded by a [Filter]-[Ideal IAF] TEM with the filter kernel *u*.

**Proof**

Since *u* ∈ *ℋ*, *u*(*t*) = 〈*u*(·), *K*(·, *t*)〉 by the reproducing property of the kernel *K*(*s*, *t*). Hence, $\left(u*h\right)\left(t\right)\overset{\left(a\right)}{=}\int_{\mathbb{R}}h\left(w\right)u\left(t-w\right)dw\overset{\left(b\right)}{=}\int_{0}^{T}h\left(w\right)\int_{0}^{T}u\left(z\right)\bar{K\left(z,t-w\right)}$

$dz\;dw\overset{\left(c\right)}{=}\int_{0}^{T}u\left(z\right)\int_{0}^{T}h\left(w\right)\bar{K\left(w,t-z\right)}dw\;dz\overset{\left(d\right)}{=}\int_{0}^{T}u(z)(𝒫h)(t-z)dz$ $\overset{\left(e\right)}{=}(u*𝒫h)(t)$, where (*a*) follows from the commutativity of convolution, (*b*) from the reproducing property of the kernel *K* and the assumption that supp⁡(*h*)⊆[0, *T*], (*c*) from the equality *K*(*z*, *t* − *w*) = *K*(*w*, *t* − *z*), (*d*) from the definition of *𝒫h* in (6), and (*e*) from the definition of convolution for periodic functions [23]. It follows that on the interval *t* ∈ [0, *T*], (5) can be rewritten as

(7) $$\begin{array}{c} \int_{t_{k}}^{t_{k+1}}\left(u*𝒫h\right)\left(s\right)ds\overset{\left(f\right)}{=}\int_{t_{k}}^{t_{k+1}}\left(𝒫h*u\right)\left(s\right)ds=q_{k}, \end{array}$$

for all *k* = 1,2,…, *n* − 1, where (*f*) comes from the commutativity of convolution. The right-hand side of (7) is the *t*-transform of a [Filter]-[Ideal IAF] TEM with an input *𝒫h* and a filter that has an impulse response *u*. Hence, a TDM can identify *𝒫h*, given a filter-output pair (*u*, *𝕋*).

**Figure 3:**
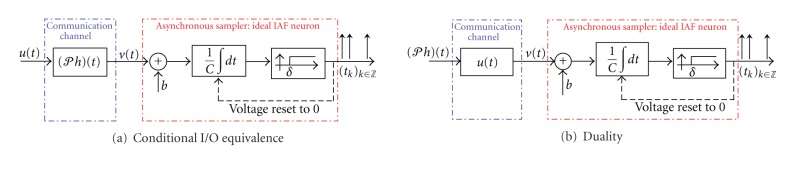
Conditional duality between channel identification and time encoding. (a) For all *u* ∈ *ℋ*, the [Filter]-[Ideal IAF] circuit with an input-filter pair (*u*, *h*) is I/O equivalent to a [Filter]-[Ideal IAF] circuit with an input-filter pair (*u*, *𝒫h*). (b) The input-filter pair (*u*, *𝒫h*) in channel identification is dual to the (*𝒫h*, *u*) pair in time encoding.

The conditional duality between time encoding and channel identification is visualized in Figure 3. First, we note the *conditional I/O equivalence* between the circuit in Figure 3(a) and the original circuit in Figure 2(a). The equivalence is conditional since *𝒫h* is a projection onto a particular space *ℋ* and the two circuits are I/O-equivalent only for input signals in that space. Second, identifying the filter of the circuit in Figure 3(a) is the same as decoding the signal encoded with the circuit in Figure 3(b). Note that the filter projection *𝒫h* is now treated as the input to the [Filter]-[Ideal IAF] circuit and the signal *u* appears as the impulse response of the filter. Effectively, we have transformed the channel identification problem into a time decoding problem and we can use the TDM machinery of [5] to identify the filter projection (*𝒫h*)(*t*) on *t* ∈ [0, *T*].

### 3.2. Channel Identification from Asynchronous Measurements

 Given the parameters of the asynchronous sampler, the measurements *q*_*k*_ of the channel output *v* can be readily computed from spike times (*t*_*k*_)_*k*=1_^*n*^ using the definition of *q*_*k*_ ((5) for the IAF neuron). Furthermore, as we will now show, for a known input signal, these measurements can be reinterpreted as measurements of the channel itself.

**Lemma 8**

There is a function *ϕ*_*k*_(*t*) = ∑_*l*=−*L*_^*L*^ *ϕ*_*l*,*k*_ *e*_*l*_(*t*) ∈ *ℋ*, such that the *t*-transform of the [Filter]-[Ideal IAF] neuron in (7) can be written as

(8) $$\begin{array}{c} \langle 𝒫h,\phi_{k}\rangle =q_{k}, \end{array}$$

and $\phi_{l,k}=\sqrt{T}\int_{t_{k}}^{t_{k+1}}{\bar{u}}_{l}{\bar{e}}_{l}(t)dt$ for all *l* = −*L*, −*L* + 1,…, *L* and *k* = 1,2,…, *n* − 1.

**Proof**

The linear functional *ℒ*_*k*_ : *ℋ* → ℝ defined by

(9) $$\begin{array}{c} {ℒ}_{k}\left(w\right)=\int_{t_{k}}^{t_{k+1}}\left(u*w\right)\left(s\right)ds, \end{array}$$

where *w* ∈ *ℋ*, is bounded. Thus, by the Riesz representation theorem [22], there exists a function *ϕ*_*k*_ ∈ *ℋ* such that *ℒ*_*k*_(*w*) = 〈*w*, *ϕ*_*k*_〉, *k* = 1,2,…, *n* − 1, and *q*_*k*_ = *ℒ*_*k*_(*𝒫h*) = ∫_*t*_*k*__^*t*_*k*+1_^(*u*∗*𝒫h*)(*s*)*ds* = 〈*𝒫h*, *ϕ*_*k*_〉. Since *ϕ*_*k*_ ∈ *ℋ*, we have *ϕ*_*k*_(*t*) = ∑_*l*=−*L*_^*L*^ *ϕ*_*l*,*k*_ *e*_*l*_ for some *ϕ*_*l*,*k*_ ∈ *ℂ*, *l* = −*L*, −*L* + 1,…, *L*. To find the latter coefficients, we note that $\phi_{l,k}=\langle \phi_{k},e_{l}\rangle =\bar{\langle e_{l},\phi_{k}\rangle }=\bar{{ℒ}_{k}(e_{l})}$. By definition of *ℒ*_*k*_ in (9), ${ℒ}_{k}(e_{l})=\int_{t_{k}}^{t_{k+1}}(u*e_{l})(t)dt=\int_{t_{k}}^{t_{k+1}}\int_{0}^{T}\sum_{i=-L}^{L}u_{i}e_{i}\left(s\right)e_{l}\left(t-s\right)ds\;dt=\sqrt{T}\int_{t_{k}}^{t_{k+1}}u_{l}e_{l}(t)dt$.

Since *q*_*k*_ = ∫_*t*_*k*__^*t*_*k*+1_^(*u*∗*𝒫h*)(*s*)*ds* = 〈*v*, *𝒫*1_[*t*_*k*_,*t*_*k*+1_]_〉, the measurements *q*_*k*_ are projections of *v* = *u*∗*𝒫h* onto *𝒫*1_[*t*_*k*_,*t*_*k*+1_]_, *k* = 1,2,…, *n* − 1. Assuming that *u* is known and there are enough measurements available, *𝒫h* can be obtained by first recovering *v* from these projections and then deconvolving it with *u*. However, this two-step procedure does not work when the circuit is not producing enough measurements and one cannot recover *v*. A more direct route is suggested by Lemma 8, since the measurements (*q*_*k*_)_*k*=1_^*n*−1^ can also be interpreted as the projections of *𝒫h* onto *ϕ*_*k*_, that is, 〈*𝒫h*, *ϕ*_*k*_〉, *k* = 1,2,…, *n* − 1. A natural question then is how to identify *𝒫h* directly from the latter projections.

**Lemma 9**

Let *u* ∈ *ℋ* be the input to a [Filter]-[Ideal IAF] circuit with *h* ∈ *H*. If the number of spikes *n* generated by the neuron in a time interval of length *T* satisfies *n* ≥ 2*L* + 2, then the filter projection *𝒫h* can be perfectly identified from the I/O pair (*u*, *𝕋*) as (*𝒫h*)(*t*) = ∑_*l*=−*L*_^*L*^ *h*_*l*_ *e*_*l*_(*t*), where **h** = Φ^+^ **q** with [**q**]_*k*_ = *q*_*k*_ and Φ^+^ denotes the Moore-Penrose pseudoinverse of Φ. The matrix Φ is of size (*n* − 1)×(2*L* + 1) and its elements are given by

(10) $$\begin{array}{c} {\left[\Phi \right]}_{kl}=\{\begin{array}{cc} u_{l}\left(t_{k+1}-t_{k}\right), & l=0,\\ \frac{u_{l}L\sqrt{T}\left(e_{l}\left(t_{k+1}\right)-e_{l}\left(t_{k}\right)\right)}{jl\Omega }, & l\neq 0. \end{array}\;\; \end{array}$$

**Proof**

Since *𝒫h* ∈ *ℋ*, it can be written as (*𝒫h*)(*t*) = ∑_*l*=−*L*_^*L*^ *h*_*l*_ *e*_*l*_(*t*). Then from (8) we have

(11) $$\begin{array}{c} q_{k}=\langle 𝒫h,\phi_{k}\rangle =\sum_{l=-L}^{L}h_{l}\bar{\phi_{l,k}}. \end{array}$$

Writing (11) for all *k* = 1,2,…, *n* − 1, we obtain **q** = Φ**h** with [**q**]_*k*_ = *q*_*k*_, ${\left[\Phi \right]}_{kl}=\bar{\phi_{l,k}}$ and [**h**]_*l*_ = *h*_*l*_. This system of linear equations can be solved for **h**, provided that the rank *r*(Φ) of the matrix Φ satisfies *r*(Φ) = 2*L* + 1. A necessary condition for the latter is that the number of measurements *q*_*k*_ is at least 2*L* + 1, or, equivalently, the number of spikes *n* ≥ 2*L* + 2. Under this condition, the solution can be computed as **h** = Φ^+^ **q**.

**Remark 10**

If the signal *u* is fed directly into the neuron, then ∫_*t*_*k*__^*t*_*k*+1_^(*u*∗*𝒫h*)(*t*)*dt* = ∫_*t*_*k*__^*t*_*k*+1_^ *u*(*t*)*dt*, for *k* = 1,2,…, *n* − 1, that is, (*𝒫h*)(*t*) = *K*(*t*, 0), *t* ∈ ℝ. In other words, if there is no processing on the input signal *u*, then the kernel *K*(*t*, 0) in *ℋ* is identified as the filter projection. This is also illustrated in Figure 7.

In order to ensure that the neuron produces at least 2*L* + 1 measurements in a time interval of length *T*, it suffices to have *t*_*k*+1_ − *t*_*k*_ ≤ *T*/(2*L* + 2). Since *t*_*k*+1_ − *t*_*k*_ ≤ *Cδ*/(*b* − *c*) for |*v*(*t*)| ≤ *c* < *b*, it suffices to have *Cδ* < (*b* − *c*)*T*/(2*L* + 2). Using the definition of *T* = 2*πL*/*Ω* and taking the limit as *L* → *∞*, we obtain the familiar Nyquist-type criterion *Cδ* < *π*(*b* − *c*)/*Ω* for a bandlimited stimulus *u* ∈ *Ξ* [4, 20] (see also Section 5.1).

Ideally, we would like to identify the impulse response of the filter *h*. Note that unlike *h* ∈ *H*, the projection *𝒫h* belongs to the space *ℋ*. Nevertheless, under quite natural conditions on *h* (see Section 3.4), *𝒫h* approximates *h* arbitrarily closely on *t* ∈ [0, *T*], provided that both the bandwidth and the order of the signal *u* are sufficiently large (see also Figure 9).

The requirement of Lemma 9 that the number of spikes *n* produced by the system in Figure 2(a) has to satisfy *n* ≥ 2*L* + 2 is quite stringent and may be hard to meet in practice, especially if the order *L* of the space *ℋ* is high. In that case we have the following result.

**Theorem 11 (SISO channel identification machine)**

Let {*u* ^*i*^ | *u* ^*i*^ ∈ *ℋ*}_*i*=1_^*N*^ be a collection of *N* linearly independent stimuli at the input to a [Filter]-[Ideal IAF] circuit with *h* ∈ *H*. If the total number of spikes *n* = ∑_*i*=1_^*N*^ *n* ^*i*^ generated by the neuron satisfies *n* ≥ 2*L* + *N* + 1, then the filter projection *𝒫h* can be perfectly identified from a collection of I/O pairs {(*u* ^*i*^, *𝕋* ^*i*^)}_*i*=1_^*N*^ as

(12) $$\begin{array}{c} \left(𝒫h\right)\left(t\right)=\sum_{l=-L}^{L}h_{l}e_{l}\left(t\right), \end{array}$$

where **h** = Φ^+^ **q**. Furthermore, Φ = [Φ^1^; Φ^2^; …; Φ^*N*^], **q** = [**q** ^1^; **q** ^2^; …; **q** ^*N*^] and [**q** ^*i*^]_*k*_ = *q*_*k*_^*i*^ with each Φ^*i*^ of size (*n* ^*i*^ − 1)×(2*L* + 1) and **q** ^*i*^ of size (*n* ^*i*^ − 1) × 1. The elements of matrices Φ^*i*^ are given by

(13) $$\begin{array}{c} {\left[\Phi^{i}\right]}_{kl}=\{\begin{array}{cc} u_{l}^{i}\left(t_{k+1}^{i}-t_{k}^{i}\right), & l=0,\\ \frac{u_{l}^{i}L\sqrt{T}\left(e_{l}\left(t_{k+1}^{i}\right)-e_{l}\left(t_{k}^{i}\right)\right)}{jl\Omega }, & l\neq 0, \end{array}\;\; \end{array}$$

for all *k* = 1,2,…, *n* − 1, *l* = −*L*, −*L* + 1,…, *L*, and *i* = 1,2,…, *N*.

**Proof**

Since *𝒫h* ∈ *ℋ*, it can be written as (*𝒫h*)(*t*) = ∑_*l*=−*L*_^*L*^ *h*_*l*_ *e*_*l*_(*t*). Furthermore, since the stimuli are linearly independent, the measurements (*q*_*k*_^*i*^)_*k*=1_^*n*^*i*^−1^ provided by the IAF neuron are distinct. Writing (5) for a stimulus *u* ^*i*^, we obtain

(14) $$\begin{array}{c} q_{k}^{i}=\langle 𝒫h,\phi_{k}^{i}\rangle =\sum_{l=-L}^{L}h_{l}\bar{\phi_{l,k}^{i}}, \end{array}$$

or **q** ^*i*^ = Φ^*i*^ **h**, with [**q** ^*i*^]_*k*_ = *q*_*k*_^*i*^, ${\left[\Phi^{i}\right]}_{kl}=\bar{\phi_{l,k}^{i}}$ and [**h**]_*l*_ = *h*_*l*_. Repeating for all *i* = 1,…, *N*, we get **q** = Φ**h** with Φ = [Φ^1^; Φ^2^; …; Φ^*N*^] and **q** = [**q** ^1^; **q** ^2^; …; **q** ^*N*^]. This system of linear equations can be solved for **h**, provided that the rank *r*(Φ) of matrix Φ satisfies *r*(Φ) = 2*L* + 1. A necessary condition for the latter is that the total number *n* = ∑_*i*=1_^*N*^ *n* ^*i*^ of spikes generated in response to all *N* signals satisfies *n* ≥ 2*L* + *N* + 1. Then, the solution can be computed as **h** = Φ^+^ **q**. To find the coefficients $\bar{\phi_{l,k}^{i}}$, we note that $\phi_{l,k}^{i}=\bar{{ℒ}_{k}^{i}(e_{l})}$ (see Lemma 8). Hence, the result follows.

**Figure 4:**
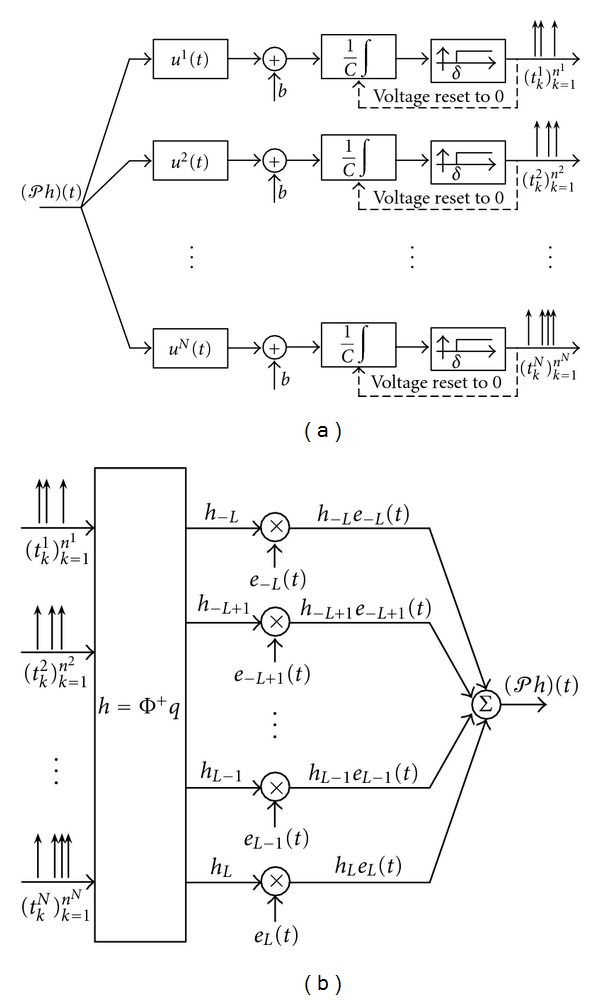
SISO CIM algorithm for the [Filter]-[Ideal IAF] circuit. (a) Time encoding interpretation of the channel identification problem. (b) Block diagram of the SISO channel identification machine.

The time encoding interpretation of the channel identification problem for a SISO [Filter]-[Ideal IAF] circuit is shown in Figure 4(a). The block diagram of the SISO CIM in Theorem 11 is shown in Figure 4(b). Note that the key idea behind the SISO CIM is the introduction of multiple linearly independent test signals *u* ^*i*^ ∈ *ℋ*, *i* = 1,2, ..., *N*. When the [Filter]-[Ideal IAF] circuit is producing very few measurements of *𝒫h* in response to any given test signal *u* ^*i*^, we use more signals to obtain additional measurements. We can do so and identify *𝒫h* because *𝒫h* ∈ *ℋ* is fixed. In contrast, identifying *𝒫h* in a two-step deconvolving procedure requires reconstructing at least one *v* ^*i*^. This is an ill-posed problem since each *v* ^*i*^ is signal-dependent and has a small number of associated measurements.

### 3.3. Examples

We now demonstrate the performance of the identification algorithms in Lemma 9 and Theorem 11. First, we identify a filter in the SISO [Filter]-[Ideal IAF] circuit (Figure 2(a)) from a single I/O pair when this circuit produces a sufficient number of measurements in an interval of length *T*. Second, we identify the filter using multiple I/O pairs for the case when the number of measurements produced in response to any given input signal is small. Finally, we consider the SISO [Filter]-[Nonlinear Oscillator-ZCD] circuit with multiplicative coupling (Figure 2(b)) and identify its filter from multiple I/O pairs.

#### 3.3.1. SISO [Filter]-[Ideal IAF] Circuit, Single I/O Pair

**Figure 5:**
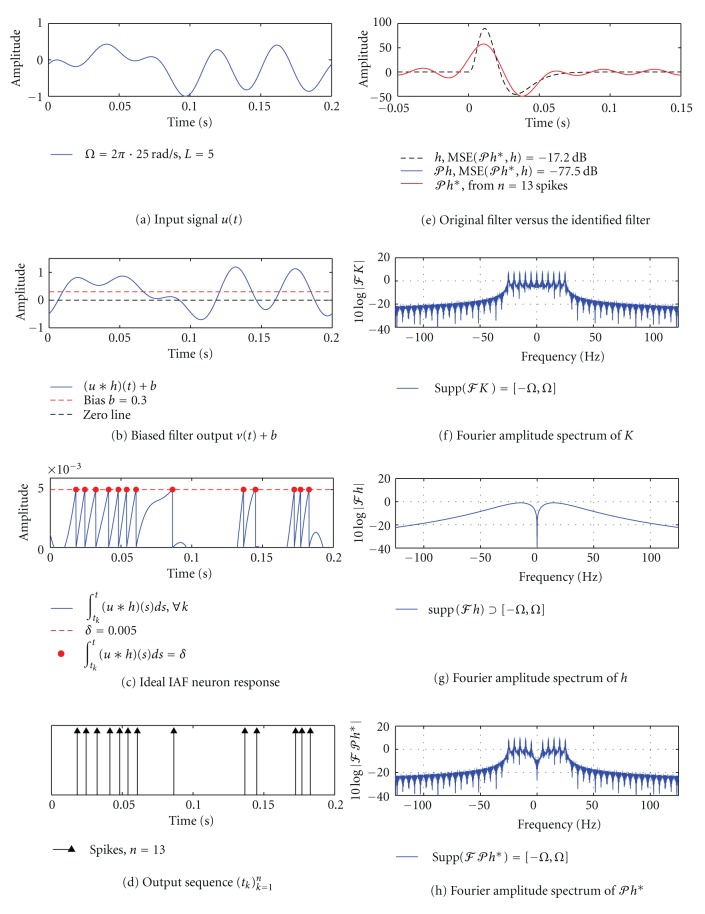
Channel identification in a SISO [Filter]-[Ideal IAF] circuit using a single I/O pair. (a) An input signal *u* is bandlimited to 25 Hz. The order of the space is *L* = 5. (b) The corresponding biased output of the filter *v*(*t*) + *b*. (c) The filter output in (b) is integrated by the ideal IAF neuron. Whenever the membrane potential reaches a threshold *δ*, a spike is produced by the neuron and the potential is reset to 0. (d) The neuron generated a total of 13 spikes. (e) The identified impulse response of the filter 𝒫h* (red) is shown together with the original filter *h* (dashed black) and its projection *𝒫h* (blue). The MSE between 𝒫h* and *𝒫h* is −77.5 dB. (f)–(h) Fourier amplitude spectra of *K*, *h* and 𝒫h*. Note that supp⁡(*ℱK*) = supp⁡(*ℱ* 𝒫h*) = [−*Ω*, *Ω*] but supp⁡(*ℱh*)⊃[−*Ω*, *Ω*]. In other words, 𝒫h* ∈ *ℋ* but *h* ∉ *ℋ*.

We model the dendritic processing filter using the causal linear kernel

(15) $$\begin{array}{c} h\left(t\right)=ce^{-\alpha t}\left[\frac{{\left(\alpha t\right)}^{3}}{3!}-\frac{{\left(\alpha t\right)}^{5}}{5!}\right],\;t\in \left[0,0.1\right]\;\text{s}, \end{array}$$

with *c* = 3 and *α* = 200. The general form of this kernel was suggested in [24] as a plausible approximation to the temporal structure of a visual receptive field. Since the length of the filter support *S* = 0.1 s, we will need to use a signal with a period *T* ≥ 0.1 s. In Figure 5(a), we apply a signal *u* that is bandlimited to 25 Hz and has a period of *T* = 0.2 s, that is, the order of the space *L* = *T* · *Ω*/(2*π*) = 5. The biased output of the filter *v* = (*u*∗*h*) + *b* is then fed into an ideal integrate-and-fire neuron (Figure 5(b)). Here the bias *b* guarantees that the output of the integrator reaches the threshold value in finite time. Whenever the biased filter output is above zero (Figure 5(b)), the membrane potential is increasing (Figure 5(c)). If the membrane potential ∫_*t*_*k*__^*t*^[(*u*∗*h*)(*s*) + *b*]*ds* reaches a threshold *δ*, a spike is generated by the neuron at a time *t*_*k*+1_ and the potential is reset to zero (Figure 5(c)). The resulting spike train (*t*_*k*_)_*k*=1_^*n*^ at the output of the [Filter]-[Ideal IAF] circuit is shown in Figure 5(d). Note that the circuit generated a total of *n* = 13 spikes in an interval of length *T* = 0.2 s. According to Theorem 14, we need at least *n* = 2*L* + 2 = 12 spikes, corresponding to 2*L* + 1 = 11 measurements, in order to identify the projection *𝒫h* of the filter *h* loss-free. Hence, for this particular example, it will suffice to use a single I/O pair (*u*, *𝕋*).

In Figure 5(e), we plot the original impulse response of the filter *h*, the filter projection *𝒫h*, and the filter 𝒫h*. The latter filter was identified using the algorithm in Theorem 14. Notice that the identified impulse response 𝒫h* (red) is quite different from *h* (dashed black). In contrast, and as expected, the blue and red curves corresponding, respectively, to *𝒫h* and 𝒫h* are indistinguishable. The mean squared error (MSE) between 𝒫h* and *𝒫h* amounts to −77.5 dB.

The difference between *𝒫h* and *h* is further evaluated in Figures 5(f)–5(h). By definition of *𝒫h* in (6), $𝒫h=h*\bar{K(\cdot ,0)}$, or *ℱ*(*𝒫h*) = *ℱ*(*h*)*ℱ*(*K*(·, 0)) since $\bar{K}=K$. Hence both the projection *𝒫h* and the identified filter 𝒫h* will contain frequencies that are present in the reproducing kernel *K*, or equivalently in the input signal *u*. In Figure 5(f) we show the double-sided Fourier amplitude spectrum of *K*(*t*, 0). As expected, we see that the kernel is bandlimited to 25 Hz and contains 2*L* + 1 = 11 distinct frequencies. On the other hand, as shown in Figure 5(g), the original filter *h* is not bandlimited (since it has a finite temporal support). As a result, the input signal *u* explores *h* in a limited spectrum of [−*Ω*, *Ω*]  rad/s, effectively projecting *h* onto the space *ℋ* with *Ω* = 2*π* · 25  rad/s and *L* = 5. The Fourier amplitude spectrum of the identified projection 𝒫h* is shown in Figure 5(h).

#### 3.3.2. SISO [Filter]-[Ideal IAF] Circuit, Multiple I/O Pairs

Next, we identify the projection of *h* onto the space of functions that are bandlimited to 100 Hz and have the same period *T* = 0.2 s as in the first example. This means that the order *L* of the space of input signals *ℋ* is *L* = *T* · *Ω*/(2*π*) = 20. In order to identify the projection *𝒫h* loss-free, the neuron has to generate at least 2*L* + 1 = 41 measurements. If the neuron produces about 13 spikes (12 measurements) on an interval of length *T*, as in the previous example, a single I/O pair will not suffice. However, we can still recover the projection *𝒫h* if we use multiple I/O pairs.

**Figure 6:**
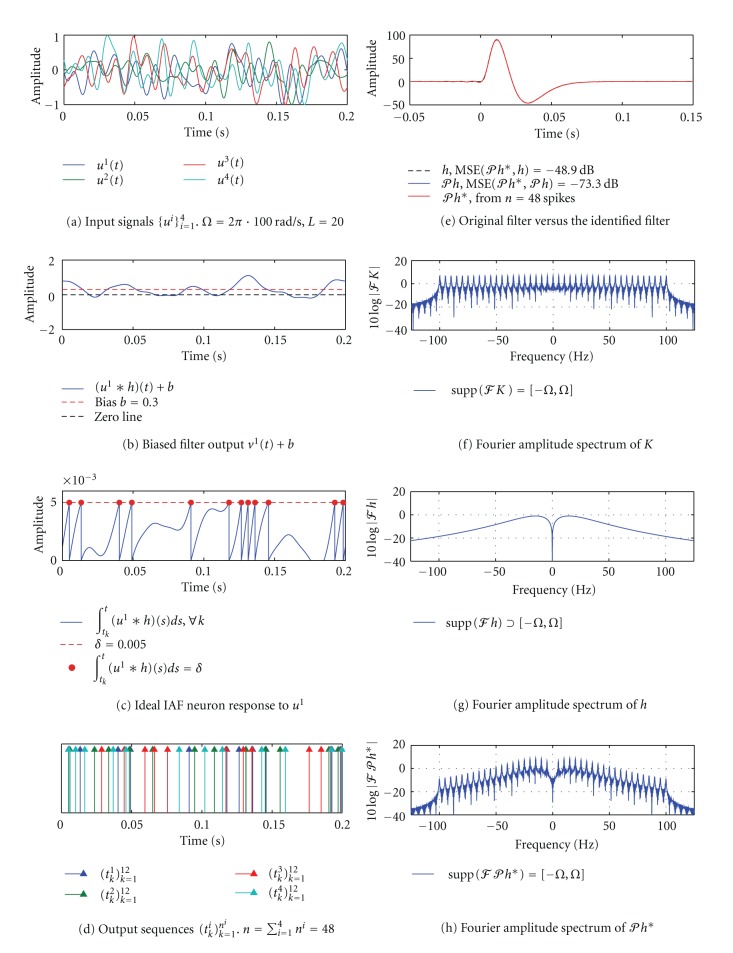
Channel identification in a SISO [Filter]-[Ideal IAF] circuit using multiple I/O pairs. (a) Input signals *u* ^1^,…, *u* ^4^ are bandlimited to 100 Hz. The order of the space *L* = 20. (b) Biased output of the filter *v* ^1^(*t*) + *b* in response to the stimulus *u* ^1^. (c) The filter output in (b) is integrated by an ideal IAF neuron. (d) The neuron generated a total of 48 spikes in response to all 4 input signals. (e) The identified impulse response 𝒫h* (red) is shown together with the original filter *h* (dashed black) and its projection *𝒫h* (blue). The MSE between 𝒫h* and *𝒫h* is −73.3 dB. (f)–(h) Fourier amplitude spectra of *K*, *h* and 𝒫h*. Note that supp⁡(*ℱK*) = [−*Ω*, *Ω*] = supp⁡(*ℱ* 𝒫h*) but supp⁡(*ℱh*)⊃[−*Ω*, *Ω*]. In other words, 𝒫h* ∈ *ℋ* but *h* ∉ *ℋ*.

In Figure 6 we illustrate identification of the filter using the algorithm in Theorem 11. A total of 48 spikes were produced by the neuron in response to four different signals *u* ^1^,…  , *u* ^4^. Since 48 > 2*L* + *N* + 1 = 45, the MSE between the identified filter 𝒫h* (red) and the projection *𝒫h* (blue) is −73.3 dB.

#### 3.3.3. SISO [Filter]-[Ideal IAF] Circuit, *h*(*t*) = *δ*(*t*)

**Figure 7:**
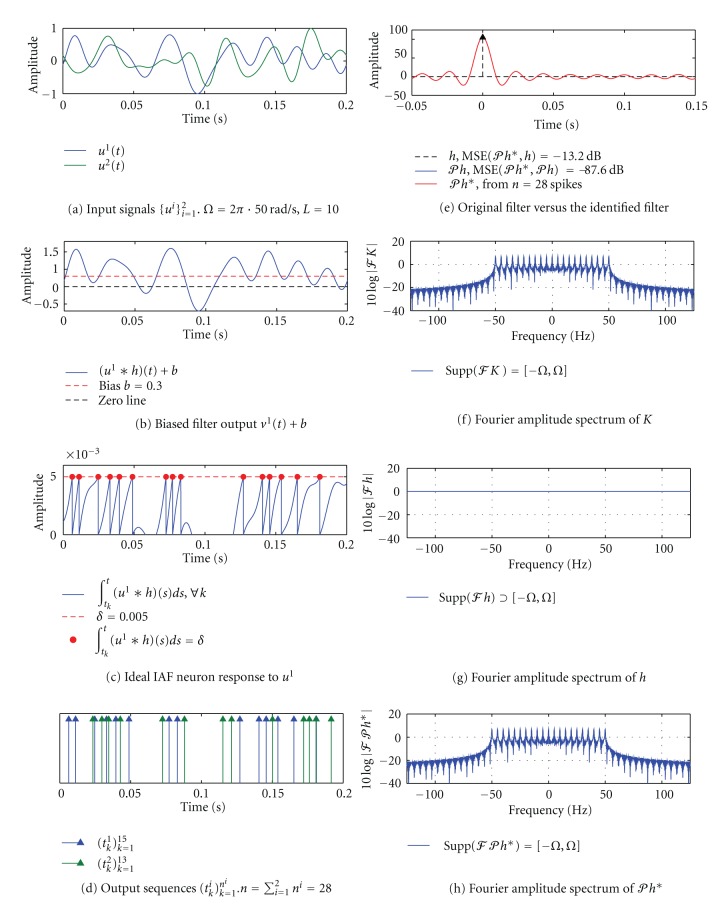
Channel identification for *h*(*t*) = *δ*(*t*). (a) Input signals *u* ^1^, *u* ^2^ are bandlimited to 50 Hz. The order of the space *L* = 10. (b) Biased output of the filter *v* ^1^(*t*) + *b* in response to the stimulus *u* ^1^. (c) The filter output in (b) is integrated by an ideal IAF neuron. (d) The neuron generated a total of 28 spikes in response to 2 input signals. (e) The identified filter 𝒫h* (red) is exactly the kernel *K*(*t*, 0) for *ℋ*_*Ω*,*L*_^1^ with *Ω* = 2*π* · 10  rad/s and *L* = 10. Also shown is the original filter *h* = *δ* (dashed black) and its projection $𝒫h=\delta *\bar{K(\cdot ,0)}=K(\cdot ,0)$ (blue). The MSE between 𝒫h* and *𝒫h* is −87.6 dB. (f)–(h) Fourier amplitude spectra of *K*, *h* and 𝒫h*. As before, 𝒫h* ∈ *ℋ* but *h* ∉ *ℋ*.

Now we consider a special case when the channel does not alter the input signal, that is, when *h*(*t*) = *δ*(*t*), *t* ∈ ℝ, is the Dirac delta function. As explained in Remark 10, the CIM should identify the projection of *δ*(*t*) onto *ℋ*, that is, it should identify the kernel *K*(*t*, 0). This is indeed the case as shown in Figure 7.

#### 3.3.4. SISO [Filter]-[Nonlinear Oscillator-ZCD] Circuit, Multiple I/O Pairs

Next we consider a SISO circuit consisting of a channel in cascade with a nonlinear dynamical system that has a stable limit cycle. We assume that the (positive) output of the channel *v*(*t*) + *b* is multiplicatively coupled to the dynamical system (Figure 2(b)) so that the circuit is governed by a set of equations

(16) $$\begin{array}{c} \frac{dy}{dt}=\left(v\left(t\right)+b\right)f\left(y\right). \end{array}$$

A system (16) followed by a zero-crossing detector is an example of a TEM with multiplicative coupling and has been previously investigated in [6]. It can be shown that such a TEM is input/output equivalent to an IAF neuron with a threshold *δ* that is equal to the period of the dynamical system on a stable limit cycle [6].

As an example, we consider a [Filter]-[van der Pol - ZCD] TEM with the van der Pol oscillator described by a set of equations

(17) $$\begin{array}{c} \frac{dy_{1}}{dt}=\left(u*h+b\right)\left[\mu \left(y_{1}-\frac{1}{3}y_{1}^{3}\right)-y_{2}\right],\\ \frac{dy_{2}}{dt}=\left(u*h+b\right)y_{1}, \end{array}$$

where *μ* is the damping coefficient [6]. We assume that *y*_1_ is the only observable state of the oscillator and without loss of generality we choose the zero phase of the limit cycle to be the peak of *y*_1_.

**Figure 8:**
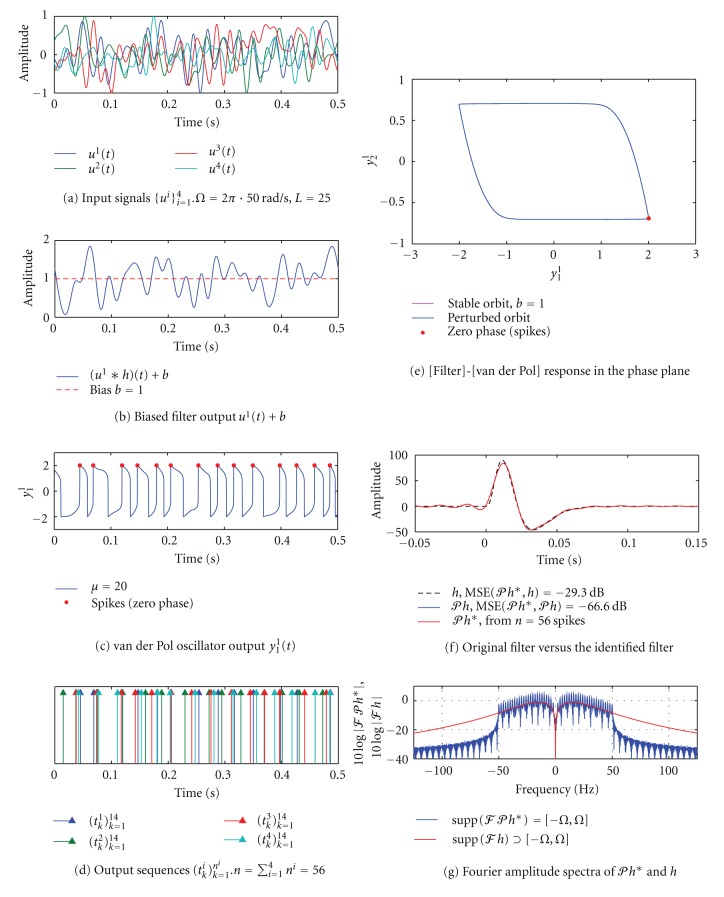
Channel identification in a SISO [Filter]-[van der Pol-ZCD] circuit using multiple I/O pairs. (a) Input signals *u* ^1^,…, *u* ^4^ are bandlimited to 50 Hz. The order of the space *L* = 25. (b) Biased output of the filter *v* ^1^(*t*) + *b* in response to the stimulus *u* ^1^. (c) Downward and upward deviations of *v* ^1^(*t*) + *b* from the bias *b* cause the oscillator to slow down and to speed up, respectively. The damping coefficient *μ* = 20. (d) The oscillator produced a total of 56 spikes in response to 4 stimuli. Here spikes correspond to the peaks of the observable state variable *y*_1_^1^. (e) A limit cycle of the van der Pol oscillator for *μ* = 20 is shown in the phase plane. In the absence of channel output, the bias *b* resulted in a constant period of oscillation *T*(*b*) = 34.7  ms. The red dot denotes the zero phase (spike) of an oscillation. (f) The identified filter 𝒫h* (red) is shown together with the original filter *h* (dashed black) and its projection *𝒫h* (blue). The MSE between 𝒫h* and *𝒫h* is −66.6 dB. (g) Fourier amplitude spectra of *h* and 𝒫h*. As before, 𝒫h* ∈ *ℋ* but *h* ∉ *ℋ*.

In Figure 8, we show the results of a simulation in which a SISO CIM was used to identify the channel. Input signals (Figure 8(a)) were bandlimited to 50 Hz and had a period *T* = 0.5 s, that is, *L* = 25. In the absence of an input, that is, when *u* = 0, a constant bias *b* = 1 (Figure 8(b)) resulted a in period of 34.7 ms on a stable limit cycle (Figure 8(e)). As seen in Figures 8(b) and 8(c), downward/upward deviations of *v* ^1^(*t*) + *b* in response to *u* ^1^ resulted in the slowing-down/speeding-up of the oscillator. In order to identify the filter projection onto a space of order *L* = 25 loss-free, we used a total of *n* = 56 zeros at the output of the zero-crossing detector (Figure 8(d)). This is 1 more zero than the rank requirement of 2*L* + *N* + 1 = 55 zeros, or equivalently of 2*L* + 1 = 51 measurements. The MSE between the identified filter 𝒫h* (red) and the projection *𝒫h* (blue) is −66.6 dB.

### 3.4. Convergence of the SISO CIM Estimate

 Recall, that the original problem of interest is that of recovering the impulse response of the filter *h*. The CIM lets us identify the projection *𝒫h* of that filter onto the input space. A natural question to ask is whether *𝒫h* converges to *h* and if so how and under what conditions. We formalize this below.

**Proposition 12**

If ∫_0_^*T*^|*h*(*t*)|^2^ *dt* < *∞*, then *𝒫h* → *h* in the *L* ^2^ norm and almost everywhere on *t* ∈ [0, *T*] with increasing *Ω*, *L* and fixed *T*.

**Proof**

Let *T* = 2*πL*/*Ω* = const. Then *K*(*t*, 0) = (1/*T*)∑_*l*=−*L*_^*L*^ *e* ^(*jΩl*/*L*)*t*^ = (1/*T*)∑_*l*=−*L*_^*L*^ *e* ^(*j*2*πl*/*T*)*t*^ and by definition of *𝒫h* in (6), we have

(18) (𝒫h)(t)=∫0T[1T∑l=−LLe(j2πl/T)(t−s)]h(s)ds=∑l=−LL[1T∫0Th(s)e−(j2πl/T)sds]e(j2πl/T)t=∑l=−LLh^(l)e(j2πl/T)t=SLh(t),

where *S*_*L*_^*h*^ is the *L*th partial sum of the Fourier series of *h* and $\hat{h}(l)$ is the *l*th Fourier coefficient. Hence the problem of convergence of *𝒫h* to *h* is the same as that of the convergence of the Fourier series of *h*. We thus have convergence in the *L* ^2^ norm and convergence almost everywhere follows from Carleson's theorem [23].

**Remark 13**

More generally, if ∫_0_^*T*^|*h*(*t*)|^*p*^ *dt* < *∞*, *p* ∈ (1, *∞*), then *𝒫h* → *h* in the *L* ^*p*^ norm and almost everywhere by Hunt's theorem [23].

**Figure 9:**
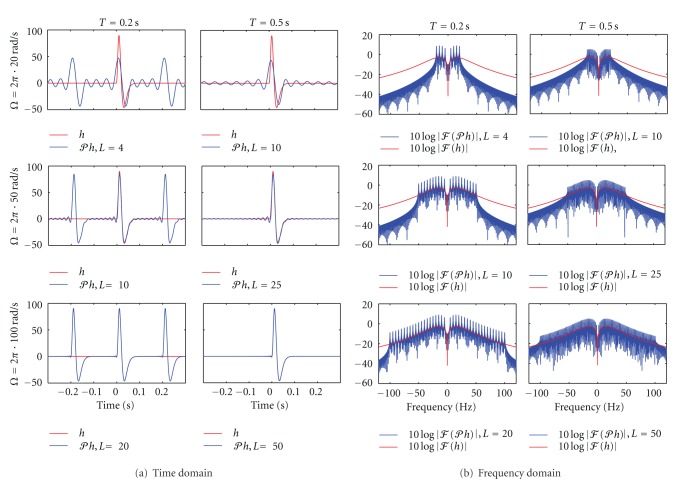
Comparison between *h* and *𝒫h* in time and frequency domains. (a) *h* (red) and its projection *𝒫h* (blue) are shown for several values of *Ω* and *L* in the time domain. *Ω* = 2*π* · 20  rad/s, 2*π* · 50  rad/s and 2*π* · 100  rad/s in the top, middle and bottom row, respectively. The period *T* is fixed at *T* = 0.2 s in the left column and *T* = 0.5 s in the right column. (b) Fourier amplitude spectra of *h* (red) and *𝒫h* (blue) for the same values of *Ω* and *L* as in (a). Note that the differentiating filter *h* clearly removes the zero-frequency (dc) coefficient corresponding to *l* = 0 in all cases.

It follows from Proposition 12 that *𝒫h* approximates *h* arbitrarily closely (in the *L* ^2^ norm, or MSE sense), given an appropriate choice of *Ω* and *L*. Since the number of measurements needed to identify the projection *𝒫h* increases linearly with *L*, a single channel identification problem leads us to consider a countably infinite number of time encoding problems in order to identify the impulse response of the filter with arbitrary precision. To provide further intuition about the relationship between *h* and *𝒫h*, we compare the two in time and frequency domains for multiple values of *Ω* and *L* in Figure 9.

## 4. MISO Channel Identification Machines

 In this section we consider the identification of a bank of *M* filters with impulse responses *h* ^*m*^, *m* = 1,2,…, *M*. We present a MISO CIM algorithm in Section 4.1, followed by an example demonstrating its performance in Section 4.2. 

### 4.1. An Identification Algorithm for MISO Channels

Consider now the MISO ASDM-based circuit in Figure 2(c), where the signal **u** = [*u* ^1^(*t*), *u* ^2^(*t*),…, *u* ^*M*^(*t*)]^*T*^, *t* ∈ [0, *T*], *M* ∈ *ℕ*, is transformed into the time sequence (*t*_*k*_)_*k*=1_^*n*^. This circuit is also an instance of a TEM and (assuming *z*(*t*_1_) = *b*) its *t*-transform is given by

(19) $$\begin{array}{c} \int_{t_{k}}^{t_{k+1}}\sum_{m=1}^{M}\left(u^{m}*h^{m}\right)\left(s\right)ds=\langle v,\phi_{k}\rangle =q_{k}, \end{array}$$

where *v* = ∑_*m*_(*u* ^*m*^∗*h* ^*m*^)(*t*), *ϕ*_*k*_ ∈ *ℋ* with *ϕ*_*k*_ = ∑_*l*_ *ϕ*_*l*,*k*_ *e*_*l*_(*t*) and *q*_*k*_ = (−1)^*k*^[2*Cδ* − *b*(*t*_*k*+1_ − *t*_*k*_)]. One simple way to identify filters *h* ^*m*^, *m* = 1,2,…, *M*, is to identify them one by one as in Theorem 11. For instance, this can be achieved by applying signals of the form **u** = [0,…, 0, *u* ^*m*^, 0,…, 0] when identifying the filter *h* ^*m*^. In a number of applications, most notably in early olfaction [25], this model of system identification cannot be applied. An alternative procedure that allows to identify all filters at once is given below.

**Theorem 14 (MISO channel identification machine)**

Let {**u** ^*i*^ | **u** ^*i*^ ∈ *ℋ* ^*M*^}_*i*=1_^*N*^ be a collection of *N* linearly-independent vector-valued signals at the input of a MISO [Filter]-[ASDM-ZCD] circuit with filters *h* ^*m*^ ∈ *H*, *m* = 1,…, *M*. The filter projections *𝒫h* ^*m*^ can be perfectly identified from a collection of I/O pairs {(**u** ^*i*^, *𝕋* ^*i*^)}_*i*=1_^*N*^ as

(20) $$\begin{array}{c} \left(𝒫h^{m}\right)\left(t\right)=\sum_{l=-L}^{L}h_{l}^{m}e_{l}\left(t\right), \end{array}$$

*m* = 1,…, *M*. Here the coefficients *h*_*l*_^*m*^ are given by **h** = Φ^+^ **q** with **q** = [**q** ^1^, **q** ^2^,…, **q** ^*N*^]^*T*^, [**q** ^*i*^]_*k*_ = *q*_*k*_^*i*^ and **h** = [*h*_−*L*_^1^,…, *h*_−*L*_^*M*^, *h*_−*L*+1_^1^,…, *h*_−*L*+1_^*M*^,…, *h*_*L*_^1^,…, *h*_*L*_^*M*^]^*T*^, provided that the matrix Φ has rank *r*(Φ) = *M*(2*L* + 1). The matrix Φ is given by

(21) Φ=[Φ10⋯00Φ2⋯0⋮⋮⋱⋮00⋯ΦN][U1U2⋮UN], with Ui=[u−Li0⋯00u−L+1i⋯0⋮⋮⋱⋮00⋯uLi],

where **u**_*l*_^*i*^ = [*u*_*l*_^*i*1^, *u*_*l*_^*i*2^,…, *u*_*l*_^*iM*^], *i* = 1,2,…, *N*. Finally, the elements of matrix Φ^*i*^ are given by

(22) $$\begin{array}{c} {\left[\Phi^{i}\right]}_{kl}=\{\begin{array}{cc} \left(t_{k+1}^{i}-t_{k}^{i}\right), & l=0,\\ \frac{L\sqrt{T}\left(e_{l}\left(t_{k+1}^{i}\right)-e_{l}\left(t_{k}^{i}\right)\right)}{jl\Omega }, & l\neq 0. \end{array}\;\; \end{array}$$

**Proof**

Since *𝒫h* ^*m*^ ∈ *ℋ* for all *m* = 1,…, *M*, it can be written as (*𝒫h* ^*m*^)(*t*) = ∑_*l*=−*L*_^*L*^ *h*_*l*_^*m*^ *e*_*l*_(*t*). Hence, for the *m*th component of the stimulus **u** ^*i*^ we get $\left(u^{im}*h^{m})(t)=(u^{im}*𝒫h^{m})(t)=\sqrt{T}\sum_{l=-L}^{L}h_{l}^{m}u_{l}^{im}e_{l}(t\right)$ and

(23) $$\begin{array}{c} v^{i}\left(t\right)=\sum_{m=1}^{M}\sqrt{T}\sum_{l=-L}^{L}h_{l}^{m}u_{l}^{im}e_{l}\left(t\right). \end{array}$$

Using the definition of *ϕ*_*k*_^*i*^ = ∑_*l*=−*L*_^*L*^ *ϕ*_*l*,*k*_^*i*^ *e*_*l*_(*t*) and substituting (23) into the *t*-transform (19), we obtain

(24) $$\begin{array}{c} q_{k}^{i}=\langle v^{i},\phi_{k}^{i}\rangle =\sum_{m=1}^{M}\sum_{l=-L}^{L}\sqrt{T}‍h_{l}^{m}u_{l}^{im}\bar{\phi_{l,k}^{i}}, \end{array}$$

or **q** ^*i*^ = Φ^*i*^ **U** ^*i*^ **h** with [**q** ^*i*^]_*k*_ = *q*_*k*_^*i*^, ${\left[\Phi^{i}\right]}_{kl}=\sqrt{T}\cdot \bar{\phi_{l,k}^{i}}$, **U** ^*i*^ = diag⁡(**u**_−*L*_^*i*^,…, **u**_*L*_^*i*^), **u**_*l*_^*i*^ = [*u*_*l*_^*i*1^,…, *u*_*l*_^*iM*^] and **h** = [*h*_−*L*_^1^,…, *h*_−*L*_^*M*^, *h*_−*L*+1_^1^,…, *h*_−*L*+1_^*M*^,…, *h*_*L*_^1^,…, *h*_*L*_^*M*^]^*T*^. Repeating for all stimuli **u** ^*i*^, *i* = 1,…, *N*, we obtain **q** = Φ**h** with Φ as specified in (21). This system of linear equations can be solved for **h**, provided that the rank of Φ satisfies the condition *r*(Φ) = *M*(2*L* + 1). To find the coefficients $\bar{\phi_{l,k}^{i}}$, we note that $\phi_{l,k}^{i}=\bar{{ℒ}_{k}^{i}(e_{l})}$. Hence, the result follows.

**Figure 10:**
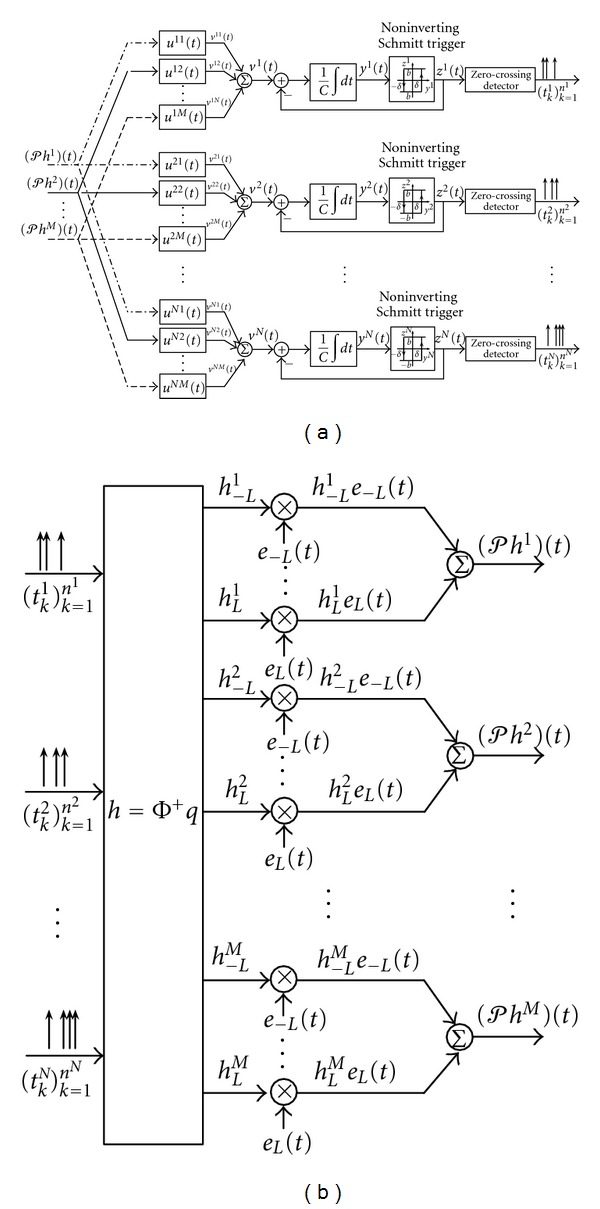
MISO CIM algorithm for the [Filter]-[ASDM-ZCD] circuit. (a) Time encoding interpretation of the MISO channel identification problem. (b) Block diagram of the MISO channel identification machine.

The MIMO time-encoding interpretation of the channel identification problem for a MISO [Filter]-[ASDM-ZCD] circuit is shown in Figure 10(a). The block diagram of the MISO CIM in Theorem 14 is shown in Figure 10(b).

**Remark 15**

From (23), we see that *v* ^*i*^ = ∑_*l*=−*L*_^*L*^ *v*_*l*_^*i*^ *e*_*l*_(*t*) with $v_{l}^{i}=\sqrt{T}\sum_{m=1}^{M}h_{l}^{m}u_{l}^{im}$. Writing this for all *i* = 1,…, *N*, we obtain **v**_*l*_ = **U**_*l*_ **h**_*l*_, where ${\left[U_{l}\right]}_{im}=\sqrt{T}u_{l}^{im}$, **h**_*l*_ = [*h*_*l*_^1^, *h*_*l*_^2^,…, *h*_*l*_^*M*^]^*T*^ and **v**_*l*_ = [*v*_*l*_^1^, *v*_*l*_^2^,…, *v*_*l*_^*N*^]^*T*^. In order to identify the multidimensional channel this system of equations must have a solution for every *l*. A necessary condition for the latter is that *N* ≥ *M*, that is, the number *N* of test signals **u** ^*i*^ is greater than the number of signal components *M*.

**Remark 16**

The rank condition *r*(Φ) = *M*(2*L* + 1) can be satisfied by increasing the number *N* of input signals **u** ^*i*^. Specifically, if on average the system is providing *ν* measurements in a time interval *t* ∈ [0, *T*], then the minimum number of test signals is *N* = ⌈*M*(2*L* + 1)/*ν*⌉.

### 4.2. Example: MISO [Filter]-[ASDM-ZCD] Circuit

 We now describe simulation results for identifying the channel in a MISO [Filter]-[ASDM - ZCD] circuit of Figure 2(c). We use three different filters:

(25) $$\begin{array}{c} h^{1}\left(t\right)=ce^{-\alpha t}\left[\frac{{\left(\alpha t\right)}^{3}}{3!}-\frac{{\left(\alpha t\right)}^{5}}{5!}\right],\\ h^{2}\left(t\right)=h^{1}\left(t-\beta \right),\\ h^{3}\left(t\right)={-h}^{1}\left(t\right), \end{array}$$

with *t* ∈ [0, 0.1] s, *c* = 3 and *α* = 200 and *β* = 20 ms. All *N* = 5 signals are bandlimited to 100 Hz and have a period of *T* = 0.2 s, that is, the order of the space *L* = 20. According to Theorem 14, the ASDM has to generate a total of at least *M*(2*L* + 1) + *N* = 128 trigger times in order to identify the projections *𝒫h* ^1^, *𝒫h* ^2^ and *𝒫h* ^3^ loss-free. We use all five triplets **u** ^*i*^ = [*u* ^*i*1^, *u* ^*i*2^, *u* ^*i*3^], *i* = 1,…, 5, to produce 131 trigger times.

**Figure 11:**
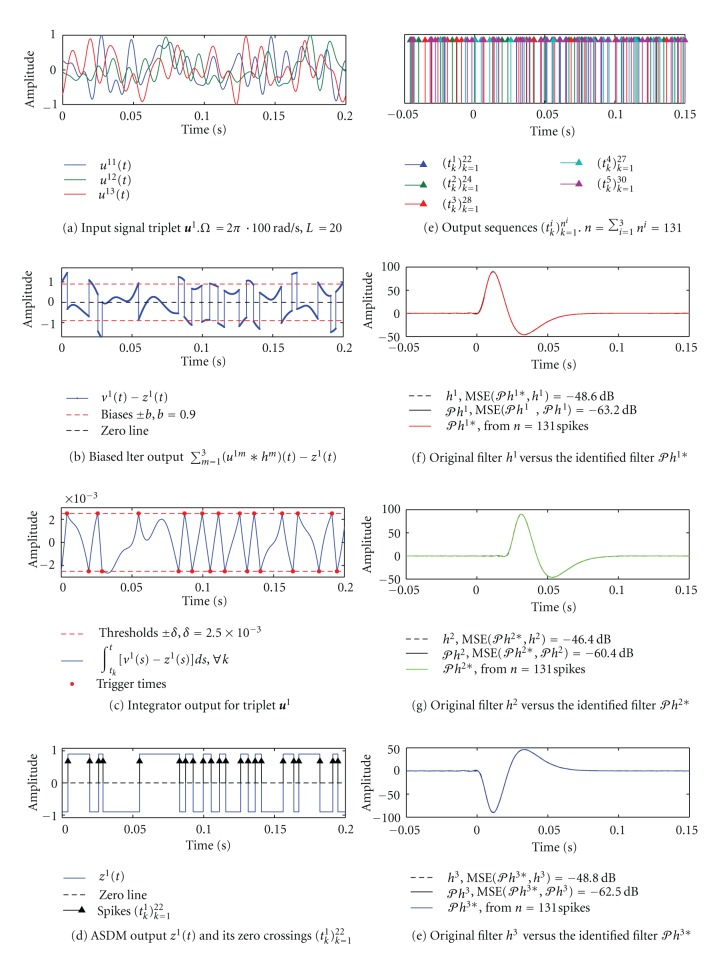
Channel identification in a MISO [FIlter]-[ASDM] circuit using multiple I/O pairs. (a) An input triplet signal **u** ^1^ = [*u* ^11^, *u* ^12^, *u* ^13^] is bandlimited to 100 Hz. The order of the space *L* = 20. (b) Biased aggregate output of the channel *v* ^1^(*t*) − *z* ^1^(*t*) in response to the triplet **u** ^1^. (c) Integrator output ∫_*t*_*k*__^*t*^[*v* ^1^(*s*) − *z* ^1^(*s*)]*ds* (blue) is compared against two thresholds +*δ* and −*δ* (dashed red). Trigger times of the noninverting Schmitt trigger are indicated by red dots. (d) The ASDM output *z* ^1^(*t*) (blue) is passed through a zero-crossing detector to produce a sequence of trigger times (*t*_*k*_^1^)_*k*=1_^22^. (e) A total of 131 trigger times were generated by the ASDM in response to five input triplets. (f)–(h) Identified filters *𝒫h* ^1∗^ (red), *𝒫h* ^2∗^ (green) and *𝒫h* ^3∗^ (blue) are shown together with the original filters *h* ^1^, *h* ^2^, *h* ^3^ (dashed black) and their projections *𝒫h* ^1^, *𝒫h* ^2^ and *𝒫h* ^3^ (black). The MSE achieved by the identification algorithm is less than −60 dB.

A single such triplet **u** ^1^ is shown in Figure 11(a). The corresponding biased aggregate channel output *v* ^1^(*t*) − *z* ^1^(*t*) is shown in Figure 11(b). Since the Schmitt trigger output *z*(*t*) switches between +*b* and −*b* (Figure 11(d)), the signal *v* ^1^(*t*) − *z* ^1^(*t*) is piece-wise continuous. Figure 11(c) shows the integrator output. Note that when *z*(*t*) = −*b*, the channel output is positively biased and the integrator output ∫_*t*_*k*__^*t*^[*v* ^1^(*s*) − *z*(*s*)]*ds* is compared against a threshold +*δ*. As soon as that threshold is reached, the Schmitt trigger output switches to *z*(*t*) = *b* and the negatively-biased channel output is compared to a threshold −*δ*. Passing the ASDM output *z* ^1^(*t*) through a zero-crossing device (Figure 11(d)), we obtain a corresponding sequence of trigger times (*t*_*k*_^1^)_*k*=1_^22^. The set of all 131 trigger times is shown in Figure 11(e). Three identified filters *𝒫h* ^1∗^, *𝒫h* ^2∗^ and *𝒫h* ^3∗^ are plotted in Figures 11(f)–11(h). The MSE between filter projections and filters recovered by the algorithm in Theorem 14 is on the order of −60 dB.

## 5. Generalizations

We shall briefly generalize the results presented in previous sections in two important directions. First, we consider a general class of signal spaces for test signals in Section 5.1. Then we discus channel models with noisy observations in Section 5.2.

### 5.1. Hilbert Spaces and RKHSs for Input Signals

 Until now we have presented channel identification results for a particular space of input signals, namely the space of trigonometric polynomials. The finite-dimensionality of this space and the simplicity of the associated inner product makes it an attractive space to work with when implementing a SISO or a MISO CIM algorithm. However, fundamentally the identification methodology relied on the the geometry of the Hilbert space of test signals [5, 26]; computational tractability was based on kernel representations in an RKHS.

**Theorem 17**

Let {*u* ^*i*^ | *u* ^*i*^ ∈ *ℋ*(*I*)}_*i*=1_^*N*^ be a collection of *N* linearly independent and bounded stimuli at the input of a [Filter]-[Asynchronous Sampler] circuit with a linear processing filter *h* ∈ *H* and the *t*-transform

(26) $$\begin{array}{c} {ℒ}_{k}^{i}\left(𝒫h\right)=q_{k}^{i}, \end{array}$$

where *ℒ*_*k*_^*i*^ : *ℋ* → ℝ is a bounded linear functional mapping *𝒫h* into a measurement *q*_*k*_^*i*^. Then there is a set of sampling functions {(*ϕ*_*k*_^*i*^)_*k*∈*ℤ*_}_*i*=1_^*N*^, in *ℋ* such that

(27) $$\begin{array}{c} q_{k}^{i}=\langle 𝒫h,\phi_{k}^{i}\rangle , \end{array}$$

for all *k* ∈ *ℤ*, *i* = 1,2,…, *N*. Furthermore, if *ℋ* is an RKHS with a kernel *K*(*s*, *t*), *s*, *t* ∈ *I*, then $\phi_{k}^{i}(t)=\bar{{ℒ}_{k}^{i}(K(\cdot ,t))}$. Let the set of representation functions {(*ψ*_*k*_^*i*^)_*k*∈*ℤ*_}_*i*=1_^*N*^, span the Hilbert space *ℋ*. Then

(28) $$\begin{array}{c} \left(𝒫h\right)\left(t\right)=\sum_{i=1\;}^{N}\sum_{k\in \mathbb{Z}}h_{k}^{i}\psi_{k}^{i}\left(t\right). \end{array}$$

Finally, if {(*ϕ*_*k*_^*i*^)_*k*∈*ℤ*_}_*i*=1_^*N*^ and {(*ψ*_*k*_^*i*^)_*k*∈*ℤ*_}_*i*=1_^*N*^ are orthogonal basis or frames for *ℋ*, then the filter coefficients amount to **h** = Φ^+^ **q**, where **h** = [**h** ^1^, **h** ^2^,…, **h** ^*N*^]^*T*^ with [**h** ^*i*^]_*k*_ = *h*_*k*_^*i*^, [Φ^*ij*^]_*lk*_ = 〈*ϕ*_*l*_^*i*^, *ψ*_*k*_^*j*^〉 and **q** = [**q** ^1^, **q** ^2^,…, **q** ^*N*^]^*T*^ with [**q** ^*i*^]_*l*_ = *q*_*k*_^*i*^  for all *i*, *j* = 1,2,…, *N*, and *k*, *l* ∈ *ℤ*.

**Proof**

By the Riesz representation theorem, since the linear functional *ℒ*_*k*_^*i*^ : *ℋ* → ℝ is bounded, there is a set of sampling functions {(*ϕ*_*k*_^*i*^)_*k*∈*ℤ*_}_*i*=1_^*N*^ in *ℋ* such that *ℒ*_*k*_^*i*^(*𝒫h*) = 〈*𝒫h*, *ϕ*_*k*_^*i*^〉. If *ℋ* is an RKHS, a sampling function *ϕ*_*k*_^*i*^ can be computed using the reproducing property of the kernel *K* as in

(29) $$\begin{array}{c} \phi_{k}^{i}\left(t\right)=\langle \phi_{k}^{i},K\left(\cdot ,t\right)\rangle \equiv \bar{\langle K(\cdot ,t),\phi_{k}^{i}\rangle }=\bar{{ℒ}_{k}^{i}\left(K\left(\cdot ,t\right)\right)}. \end{array}$$

Finally, writing all inner products 〈*ϕ*_*k*_^*i*^, *𝒫h*〉 = *q*_*k*_^*i*^ yields, with the notation above, a system of linear equations Φ**h** = **q** and the fiter coefficients amount to **h** = Φ^+^ **q**.

#### 5.1.1. Example: Paley-Wiener Space

As an example, we consider the Paley-Wiener space which is closely related to the space of trigonometric polynomials. Specifically, the finite-dimensional space *ℋ* can be thought of as a discretized version of the infinite-dimensional Paley-Wiener space

(30) $$\begin{array}{c} \Xi =\left\{u\in {𝕃}^{2}\left(\mathbb{R}\right)\;|\;\mathrm{supp}\left(ℱu\right)\subseteq \left[-\Omega ,\Omega \right]\right\} \end{array}$$

in the frequency domain. An element *u* ∈ *ℋ* has a line spectrum at frequencies *lΩ*/*L*, *l* = −*L*, −*L* + 1,…, *L*. This spectrum becomes dense in [−*Ω*, *Ω*] as *L* → *∞*. The space *Ξ* with the inner product 〈·, ·〉:*Ξ* × *Ξ* → ℝ given by

(31) $$\begin{array}{c} \langle u,w\rangle =\int_{\mathbb{R}}^{}u\left(t\right)w\left(t\right)dt \end{array}$$

is also an RKHS with an RK [22]

(32) $$\begin{array}{c} K\left(s,t\right)=\frac{\sin\left(\Omega \left(t-s\right)\right)}{\pi \left(t-s\right)}, \end{array}$$

with *t*, *s* ∈ ℝ. Defining the projection of the filter *h* onto *Ξ* as $(𝒫h)(t)=\int_{\mathbb{R}}h(s)\bar{K(s,t)}ds$, we find that Lemma 8 still holds with *ϕ*_*k*_ ∈ *Ξ* and we can extend Theorem 11 to the following.

**Proposition 18**

Let {*u* ^*i*^ | supp⁡(*ℱu* ^*i*^) = [−*Ω*, *Ω*]}_*i*=1_^*N*^ be a collection of *N* linearly independent and bounded stimuli at the input of a [Filter]-[Ideal IAF] neural circuit with a dendritic processing filter *h* ∈ *H*. If ∑_*j*=1_^*N*^(*b*/*Cδ*) > *Ω*/*π*, then (*𝒫h*)(*t*) can be perfectly identified from the collection of I/O pairs {(*u* ^*i*^, *𝕋* ^*i*^)}_*i*=1_^*N*^ as

(33) $$\begin{array}{c} \left(𝒫h\right)\left(t\right)=\sum_{i=1}^{N}\sum_{k\in \mathbb{Z}}h_{k}^{i}\psi_{k}^{i}\left(t\right), \end{array}$$

where *ψ*_*k*_^*i*^(*t*) = *K*(*t*, *t*_*k*_^*i*^), *i* = 1,2,…, *N*, and *k* ∈ *ℤ*. Finally, **h** = Φ^+^ **q**, where **h** = [**h** ^1^, **h** ^2^,…, **h** ^*N*^]^*T*^ with [**h** ^*i*^]_*k*_ = *h*_*k*_^*i*^, [Φ^*ij*^]_*lk*_ = ∫_*t*_*l*_^*i*^_^*t*_*l*+1_^*i*^^ *u* ^*i*^(*s* − *t*_*k*_^*j*^)*ds* and **q** = [**q** ^1^, **q** ^2^,…, **q** ^*N*^]^*T*^ with [**q** ^*i*^]_*l*_ = *Cδ* − *b*(*t*_*l*+1_^*i*^ − *t*_*l*_^*i*^) for all *i*, *j* = 1,2,…, *N*, and *k*, *l* ∈ *ℤ*.

**Proof**

As before, the spikes (*t*_*k*_^*i*^)_*k*∈*ℤ*_ in response to each test signal *u* ^*i*^, *i* = 1,2,…, *N*, represent distinct measurements *q*_*k*_^*i*^ = 〈*ϕ*_*k*_^*i*^, *𝒫h*〉 of (*𝒫h*)(*t*). Thus we can think of the {(*q*_*k*_^*i*^)_*k*∈*ℤ*_}_*i*=1_^*N*^'s as projections of *𝒫h* onto {(*ϕ*_*k*_^*i*^)_*k*∈*ℤ*_}_*i*=1_^*N*^, where *ϕ*_*k*_^*i*^(*t*) = *ℒ*_*k*_^*i*^(*K*(·, *t*)) = ∫_*t*_*k*_^*i*^_^*t*_*k*+1_^*i*^^∫_ℝ_ *u* ^*i*^(*z*)*K*(*s* − *z*, *t*)*dz* *ds* = ∫_*t*_*k*_^*i*^_^*t*_*k*+1_^*i*^^ *u* ^*i*^(*s* − *t*)*ds*. Since the signals are linearly independent and bounded [5], it follows that, if ∑_*i*=1_^*N*^(*b*/*Cδ*) > *Ω*/*π* or equivalently if the number of test signals *N* > *Ω* *Cδ*/*πb*, the set of functions {(*ψ*_*k*_^*i*^)_*k*∈*ℤ*_}_*i*=1_^*N*^ with *ψ*_*k*_^*i*^(*t*) = *K*(*t*, *t*_*k*_^*i*^), is a frame for *Ξ* [5, 26]. Hence

(34) $$\begin{array}{c} \left(𝒫h\right)\left(t\right)=\sum_{i=1\;}^{N}\sum_{k\in \mathbb{Z}}h_{k}^{i}\psi_{k}^{i}\left(t\right). \end{array}$$

If the set of functions {(*ϕ*_*k*_^*i*^)_*k*∈*ℤ*_}_*i*=1_^*N*^ forms a frame for *Ξ*, we can find the coefficients *h*_*k*_^*i*^, *k* ∈ *ℤ*, *i* = 1,2,…, *N*, by taking the inner product of (34) with each element of {*ϕ*_*l*_^*i*^(*t*)}_*i*=1_^*N*^ : 〈*ϕ*_*l*_^*i*^, *𝒫h*〉 = ∑_*k*∈*ℤ*_ *h*_*k*_^1^〈*ϕ*_*l*_^*i*^, *ψ*_*k*_^1^〉+∑_*k*∈*ℤ*_ *h*_*k*_^2^〈*ϕ*_*l*_^*i*^, *ψ*_*k*_^2^〉+⋯+∑_*k*∈*ℤ*_ *h*_*k*_^*N*^〈*ϕ*_*l*_^*i*^, *ψ*_*k*_^*N*^〉≡*q*_*l*_^*i*^, for *i* = 1,2,…, *N*, *l* ∈ *ℤ*. Letting [Φ^*ij*^]_*lk*_ = 〈*ϕ*_*l*_^*i*^, *ψ*_*k*_^*j*^〉, we obtain

(35) qli=∑k∈ℤ[Φi1]lkhk1+  ∑k∈ℤ[Φi2]lkhk2+⋯+∑k∈ℤ[ΦiN]lkhkN,

for *i* = 1,2,…, *N*, *l* ∈ *ℤ*. Writing (35) in matrix form, we have **q** = Φ**h** with [Φ^*ij*^]_*lk*_ = 〈*ϕ*_*l*_^*i*^, *ψ*_*k*_^*j*^〉 = 〈*ϕ*_*l*_^*i*^(·), *K*(·, *t*_*k*_^*j*^)〉 = *ϕ*_*l*_^*i*^(*t*_*k*_^*j*^) = ∫_*t*_*l*_^*i*^_^*t*_*l*+1_^*i*^^ *u* ^*i*^(*s* − *t*_*k*_^*j*^)*ds*. Finally, the coefficients *h*_*k*_^*i*^, *i* = 1,2,…, *N* and *k* ∈ *ℤ*, amount to **h** = Φ^+^ **q**.

**Figure 12:**
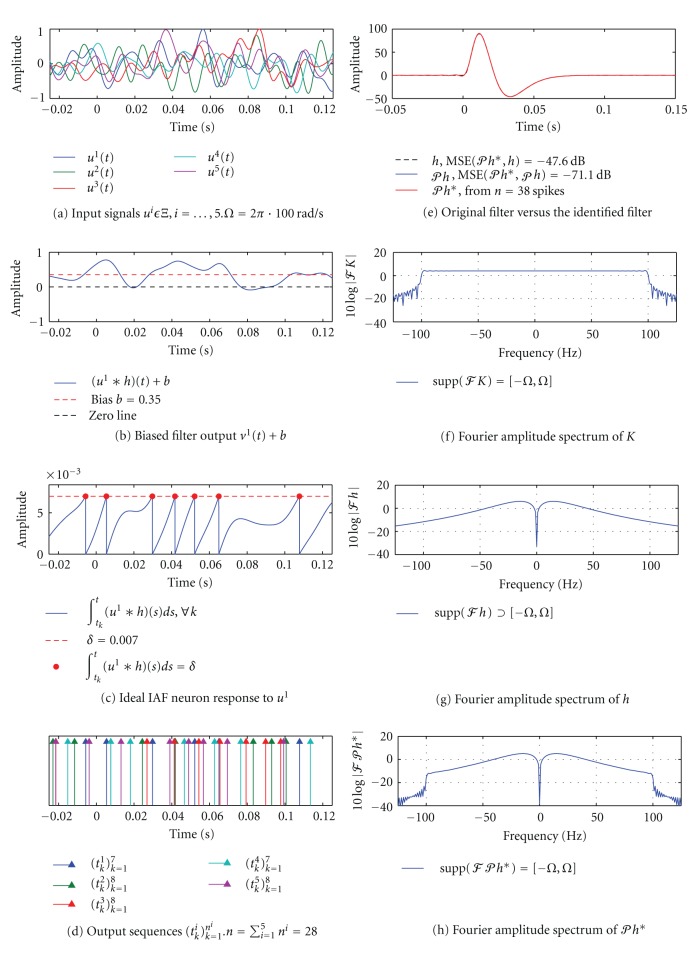
Channel identification in a SISO [Filter]-[Ideal IAF] circuit using signals from the Paley-Wiener space *Ξ*. (a) In contrast to Figure 6, input signals *u* ^*i*^ ∈ *Ξ*, *i* = 1,…  , 5. (b) Biased output of the filter *v* ^1^(*t*) + *b* in response to the stimulus *u* ^1^. (c) The filter output in (b) is integrated by an ideal IAF neuron. (d) The neuron generated a total of 38 spikes in response to all 5 input signals. (e) The identified impulse response of the filter 𝒫h* (red) is shown together with the original filter *h* (dashed black) and its projection *𝒫h* (blue). The MSE between 𝒫h* and *𝒫h* is −71.1 dB. (f)–(h) Fourier amplitude spectra of *K*, *h*, and 𝒫h*. In contrast to Figure 6, *K* and 𝒫h* do not exhibit a discrete (line) spectrum. Again, 𝒫h* ∈ *Ξ* but *h* ∉ *Ξ*.

Simulation results of a SISO CIM for a Paley-Wiener space of test signals is shown in Figure 12 Input signals *u* ^1^,…, *u* ^5^ were bandlimited to 100 Hz and the circuit generated a total of 38 spikes. The MSE between the identified filter 𝒫h* (red) and the projection *𝒫h* (blue) is −71.1 dB.

### 5.2. Channels with Noisy Observations

 In the derivations above we implicitly assumed that the I/O system was noiseless. In practice, noise is introduced either by the channel or the sampler itself. Here we revisit the *t*-transform in (5) and show that the analysis/methodology employed in the previous sections can be extended within an appropriate mathematical setting to I/O systems with noisy measurements.

Recall the *t*-transform of an ideal IAF neuron is given by ∫_*t*_*k*__^*t*_*k*+1_^(*u*∗*h*)(*t*)*dt* = 〈*𝒫h*, *ϕ*_*k*_〉 = *q*_*k*_, *k* = 1,2,…, *n* − 1, where *n* is the number of spikes generated by the neuron in an interval of length *T*. The measurements *q*_*k*_ were obtained by applying a piece-wise linear operator on the channel output *v* = *u*∗*h*. If either the channel or the sampler introduce an error, we can model it by adding a noise term *ε*_*k*_ to the *t*-transform [9]:

(36) $$\begin{array}{c} \langle 𝒫h,\phi_{k}\rangle =q_{k}+\varepsilon_{k}. \end{array}$$

Here we will assume that *ε*_*k*_ ~ *𝒩*(0, *σ* ^2^), *k* = 1,2,…, *n* − 1, are i.i.d.

In the presence of noise it is not possible to identify the projection *𝒫h* loss-free. However, we can still identify an estimate $\hat{𝒫h}$ of *𝒫h* that is optimal for an appropriately defined cost function. For example, we can formulate a bi-criterion Tikhonov regularization problem

(37) $$\begin{array}{c} \underset{\hat{𝒫h}\in ℋ}{\min}\sum_{i=1\;}^{N}\sum_{k=1}^{n-1}{\left(\langle \hat{𝒫h},\phi_{k}^{i}\rangle -q_{k}^{i}\right)}^{2}+\lambda {||\hat{𝒫h}||}_{ℋ}^{2}, \end{array}$$

where the scalar *λ* > 0 provides a trade-off between the faithfulness of the identified filter projection $\hat{𝒫h}$ to measurements (*q*_*k*_)_*k*=1_^*n*−1^ and its norm ${||\hat{𝒫h}||}_{ℋ}$.

**Theorem 19**

Problem (37) can be solved explicitly in analytical form. The optimal solution is achieved by

(38) $$\begin{array}{c} \left(\hat{𝒫h}\right)\left(t\right)=\sum_{l=-L}^{L}h_{l}e_{l}\left(t\right), \end{array}$$

with **h** = (Φ^*H*^Φ + *λ* **I**)^−1^Φ^*H*^ **q**, Φ = [Φ^1^; Φ^2^; …; Φ^*N*^] and Φ^*i*^, *i* = 1,2,…, *N*, as defined in (13).

**Proof**

Since the minimizer $\hat{𝒫h}$ is in *ℋ*, it is of the form given in (38). Substituting this into (37), we obtain

(39) $$\begin{array}{c} \underset{h\in {\mathbb{C}}^{2L+1}}{\min}{||\Phi h-q||}_{R^{n-1}}^{2}+\lambda {||h||}_{{\mathbb{C}}^{2L+1}}^{2}, \end{array}$$

where Φ = [Φ^1^; Φ^2^; …; Φ^*N*^] with Φ^*i*^, *i* = 1,2,…, *N*, as defined in (13). This quadratic optimization problem can be solved analytically by expressing the objective as a convex quadratic function *J*(**h**) = **h** ^*H*^Φ^*H*^Φ**h** − 2**q** ^*H*^Φ**h** + **q** ^*H*^ **q** + *λ* **h** ^*H*^ **h** with *H* denoting the conjugate transpose. A vector **h** minimizes *J* if and only if ∇*J* = 2(Φ^*H*^Φ + *λ* **I**)**h** − 2Φ^*H*^ **q** = 0, that is, **h** = (Φ^*H*^Φ + *λ* **I**)^−1^Φ^*H*^ **q**.

**Remark 20**

In Section 3.2, identification of the projection (*𝒫h*)(*t*) = ∑_*l*=−*L*_^*L*^ *h*_*l*_ *e*_*l*_(*t*) amounted to finding *𝒫h* ∈ *ℋ* such that the sum of the residuals (〈*𝒫h*,*ϕ*_*k*_〉−*q*_*k*_)^2^ was minimized [9]. In other words, we were solving an unconstrained convex optimization problem of the form

(40) $$\begin{array}{c} \underset{𝒫h\in ℋ}{\min}\sum_{i=1}^{N}\sum_{k=1}^{n-1}{\left(\langle 𝒫h,\phi_{k}^{i}\rangle -q_{k}^{i}\right)}^{2}\Leftrightarrow \underset{h\in {\mathbb{C}}^{2L+1}}{\min}{||\Phi h-q||}_{{\mathbb{R}}^{n-1}}^{2}, \end{array}$$

where **h** = [*h*_−*L*_,…, *h*_*L*_] and Φ = [Φ^1^; Φ^2^; …; Φ^*N*^] with Φ^*i*^, *i* = 1,2,…, *N*, as defined in (13).

#### 5.2.1. Example: Noisy SISO [Filter]-[Ideal IAF] Circuit

In the following example, we assume that noise is added to the measurements (*q*_*k*_^*i*^)_*k*=1_^*n*−1^, *i* = 1,2, by the neuron and we model that noise by introducing random thresholds that are normally distributed with a mean *δ* and a standard deviation 0.1*δ*, that is, *δ*_*k*_ ~ *𝒩*(*δ*, (0.1*δ*)^2^) :∫_*t*_*k*_^*i*^_^*t*_*k*+1_^*i*^^(*u* ^*i*^∗*h*)(*t*)*dt* = *Cδ*_*k*_ − *b*(*t*_*k*+1_^*i*^ − *t*_*k*_^*i*^) = [*Cδ* − *b*(*t*_*k*+1_^*i*^ − *t*_*k*_^*i*^)] + *C*(*δ*_*k*_ − *δ*) = *q*_*k*_^*i*^ + *ε*_*k*_^*i*^, where *ε*_*k*_^*i*^ ~ *𝒩*(0, (0.1*Cδ*)^2^). Thus random thresholds result in additive noise *ε*_*k*_^*i*^ ~ *𝒩*(0, (0.1*Cδ*)^2^), *i* = 1,2.

**Figure 13:**
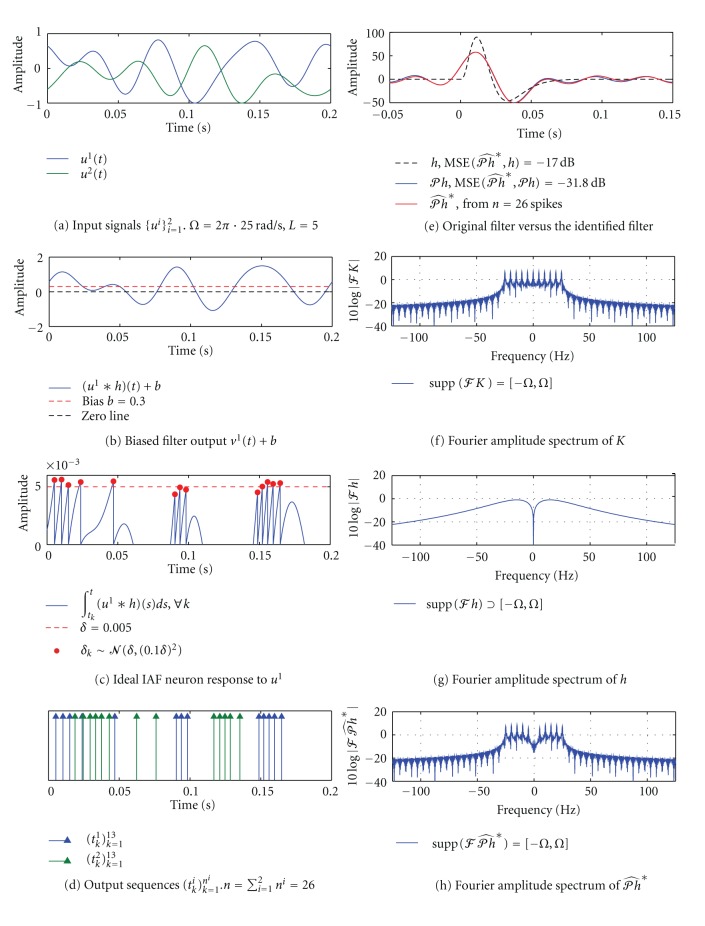
Noisy channel identification in a SISO [Filter]-[Ideal IAF] circuit using multiple I/O pairs. (a) Input signals *u* ^1^, *u* ^2^ are bandlimited to 25 Hz. The order of the space *L* = 5. (b) Biased output of the filter *v* ^1^(*t*) + *b* in response to the stimulus *u* ^1^. (c) Thresholds are random with *δ*_*k*_ ~ *𝒩*(*δ*, (0.1*δ*)^2^). (d) The neuron produced a total of 26 spikes in response to 2 stimuli. (e) The optimal estimate ${\hat{𝒫h}}^{*}$ (red) is shown together with the original filter *h* (dashed black) and its projection *𝒫h* (blue). Note that the MSE between ${\hat{𝒫h}}^{*}$ and *𝒫h* is −31.8 dB. (f)–(h) Fourier amplitude spectra of *K*, *h* and ${\hat{𝒫h}}^{*}$. As before, supp⁡(*ℱK*) = [−*Ω*, *Ω*] = $\mathrm{supp}(ℱ{\hat{𝒫h}}^{*})$ but supp⁡(*ℱh*)⊃[−*Ω*, *Ω*]. In other words, ${\hat{𝒫h}}^{*}\in ℋ$ but *h* ∉ *ℋ*.

In Figure 13(a) we show two stimuli that were used to probe the [Filter]-[Ideal IAF] circuit. Both stimuli are bandlimited to 25 Hz and have a period of *T* = 0.2 s, that is, the order of the space is *L* = 5. The response of the neuron to a biased filter output *v* ^1^(*t*) + *b* (Figure 13(b)) is shown in Figure 13(c). Note the significant deviations in thresholds *δ*_*k*_ around the mean value of *δ* = 0.05. Although a significant amount of noise is introduced into the system, we can identify an optimal estimate ${\hat{𝒫h}}^{*}$ that is still quite close to the true projection *𝒫h*. The MSE of identification is −31.8 dB.

## 6. Conclusion

In this paper we presented a class of channel identification problems arising in the context of communication channels in [Filter]-[Asynchronous Sampler] circuits. Our results are based on a key structural conditional duality result between time decoding and channel identification. The conditional duality result shows that given a class of test signals, the projection of the filter onto the space of input signals can be recovered loss-free. Moreover, the channel identification problem can be converted into a time decoding problem. We considered a number of channel identification problems that arise both in communications and in neuroscience. We presented CIM algorithms that allow one to recover projections of both one-dimensional and multi-dimensional filters in such problems and demonstrated their performance through numerical simulations. Furthermore, we showed that under natural conditions on the impulse response of the filter, the filter projection converges to the original filter almost everywhere and in the mean-squared sense (*L* ^2^ norm), with increasing bandwidth and order of the space. Thus in order to identify the impulse response of the filter with arbitrary precision, we are lead to consider a countably infinite number of time encoding problems. Finally, we generalized our results to a large class of test signal spaces and to channel models with noisy observations.
